# Covalent Proximity Inducers

**DOI:** 10.1021/acs.chemrev.4c00570

**Published:** 2024-12-18

**Authors:** Nir London

**Affiliations:** † Department of Chemical and Structural Biology, The Weizmann Institute of Science, Rehovot 7610001, Israel

## Abstract

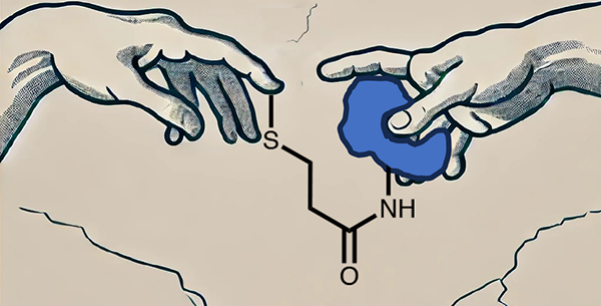

Molecules that are able to induce proximity between two proteins are finding ever increasing applications in chemical biology and drug discovery. The ability to introduce an electrophile and make such proximity inducers covalent can offer improved properties such as selectivity, potency, duration of action, and reduced molecular size. This concept has been heavily explored in the context of targeted degradation in particular for bivalent molecules, but recently, additional applications are reported in other contexts, as well as for monovalent molecular glues. This is a comprehensive review of reported covalent proximity inducers, aiming to identify common trends and current gaps in their discovery and application.

## 1 Introduction

Small molecule induced protein proximity has come a long way from the first accidental discoveries of natural products that glue various proteins^1^ to rational design of bifunctional proximity inducers that can accomplish a plethora of functions ranging from targeted degradation, through stabilization, phosphorylation, induced transcription and more^2^ culminating in dozens of new clinical compounds that are developed for a variety of indications.^3^

Inducing proximity between a target protein and a recruited effector protein has several unique advantages. The first and most obvious is that one can significantly expand the functional outcome of biological perturbation by small molecules. Whereas traditionally small molecules were used almost exclusively as inhibitors or activators (mostly for receptors), now they can be harnessed to induce the complete elimination of the target, induce various PTMs, endow an enzyme with new substrates, or induce alternative oligomeric states, to mention a few. Moreover, previously non-functional 'silent' binders, can be used for this purpose. A second important advantage is their substoichiometric activity. A single molecule of a proximity inducer can, for example, degrade multiple copies of its target protein, thereby necessitating lower concentrations to achieve a biological effect. Finally, protein proximity inducers offer the possibility of addressing “undruggable” targets. One prominent example includes cyclosporine and FK506^4^ that were discovered over 40 years ago as inhibitors of the phosphatase calcineurin, by “gluing” it to Cyclophilin A (CypA) or FKBP12 respectively. These are still being used in the clinic to date. Another example are Immunomodulatory imide drugs (IMiDs) such as thalidomide and pomalidomide that induce the degradation of the zinc-finger transcription factors Ikaros and Aiolos by gluing them to the E3 ligase Cereblon.^5,6^ Transcription factors are notoriously difficult to inhibit, as they lack small molecules binding sites, and it is only by the formation of a new molecular surface that induces this neo-protein protein interaction that enables perturbing their function. A recent similar example is the degradation of the WIZ transcription factor to induce HbF.^7^

Another modality that gained prominence in the past decade is targeted covalent inhibition.^8−11^ Covalency and in particular irreversible binding, carries its own set of advantages and challenges. Compounds that are able to form a covalent bond with their target typically display improved potency, improved selectivity (there is a strict requirement for the targeted nucleophilic residue), longer duration of action, and may confer resistance to mutations. Another major advantage of irreversible probes is the growing chemoproteomic capabilities to map out proteomic selectivity for covalent binders. Platforms such as isoTOP-ABPP,^12^ isoDTB-ABPP,^13^ TMT-ABPP^14,15^ now routinely allow the identification of molecular targets, whereas similar approaches for noncovalent compounds such as CETSA^16^ are more challenging. In terms of challenges, most electrophiles still target cysteine residues, which are relatively rare in the proteome, thereby restricting their scope. Although more and more electrophiles targeting additional residues are constantly being reported^17,18^

It therefore might not come as a surprise that many current efforts focus on the combination of these two modalities. Whether through covalent binding to the target protein or the effector (in bifunctional molecules), or by decorating known binders with electrophilic moieties to convert them into glues, we witness many recent examples of covalent proximity inducers.

**Figure 1:**
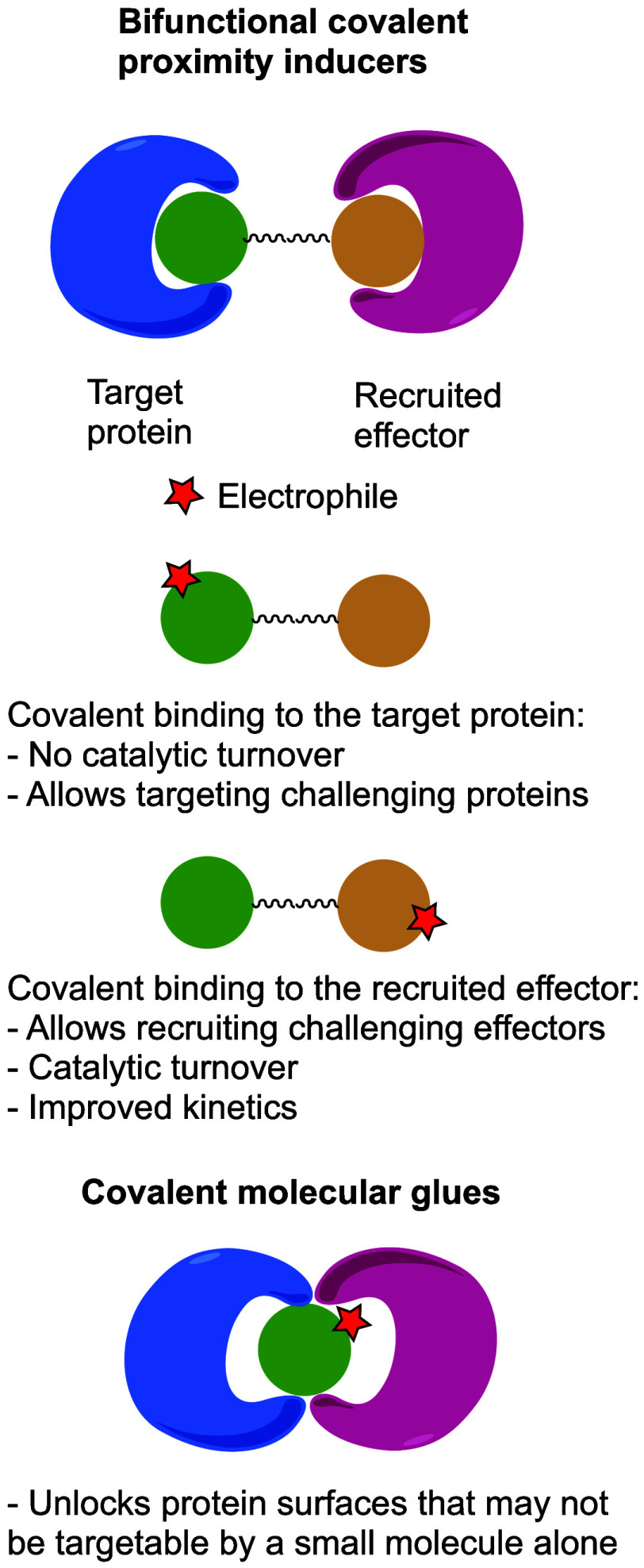
Classes of covalent proximity inducers. Covalent proximity inducers can be divided to bifunctional molecules and covalent molecular glues. Each with its advantages and disadvantages.

This review is divided into two main categories, the first describes bifunctional covalent proximity inducers, the majority of which are proteolysis targeting chimeras (PROTACs), but not exclusively. These are typically rationally designed in the sense that a covalent binder is known or discovered for either the target protein, or the recruited effector protein, and is linked to a second ligand. The second category are covalent molecular glues (or monovalent proximity inducers), historically discovered serendipitously, but recently also through rational design and through carefully designed phenotypic screens (Figure 1).

## 2 Bifunctional Covalent Proximity Inducers

By far the most prevalent bifunctional proximity inducers are PROTACs, and correspondingly, this is also the most populated class of covalent bifunctionals. However, newer modalities such as covalent deubiquitination targeting chimeras (DUBTACs) and phosphorylation inducing chimeras (PHICs) were reported, and additional modalities are expected to take advantage of covalent recruiters.

### 2.1 Covalent PROTACs

PROTACs are bifunctional, modular, molecules that bind a target protein on the one hand, typically through a target specific ligand, and a component of the ubiquitin proteasome system (UPS) on the other hand. These have been extensively reviewed.^19−21^

As PROTACs need to bind their target protein, and as some of the best binders for prototypical targets are covalent, there are many reports of covalent PROTACs that bind their target covalently. his is the focus of section 2.1.1.

Historically, and still to date, two E3 ligases have dominated the field as mediating degradation by PROTACs: Cereblon (CRBN) and von Hippel-Lindau protein (VHL). Covalent chemistry has significantly expanded the repertoire of E3 ligases and additional components of the UPS that can be recruited for targeted degradation. This is the focus of section 2.1.2.

Two important descriptors of the potency of PROTACs would be used throughout the review. DC_50_ is the concentration at which half of the target is degraded, this is typically dependent on experimental conditions such as incubation time and specific cell line. *D*_max_ is the maximal degradation that a PROTAC can incur, often referred to as the “depth” of degradation.

#### 2.1.1 Covalent Binding to the Target Protein

A major advantage of PROTACs is their substoichiometric degradation, which can allow PROTACs to be extremely potent.^22^ This poses a concern for PROTACs based on irreversible binding to the target protein, which can have only single turnover degradation. Such PROTACs could not dissociate and regenerate following the target degradation.

Several targets for which selective, high-potency, covalent inhibitors were available served as model systems for the development of covalent PROTACs. These include K-Ras^G12C^, EGFR, BTK and more recently SARS-CoV-2 main protease (M^pro^). In aggregate, these examples dispel the notion that irreversible binding is not compatible with targeted degradation. However, in most cases the direct involvement or effect of covalent binding was not directly examined. The example of K-Ras^G12C^ in particular demonstrates that covalent binding may enable degradation of targets for which there are no available high-affinity noncovalent binders. In the future, leveraging covalent binding for targets that lack classic pockets, or high-affinity binders can significantly expand the scope of targeted degradation to “undruggable” targets.

##### 2.1.1.1 K-Ras^G12C^

An early attempt to develop covalent PROTACs against K-Ras^G12C^ was based on one of the earliest K-Ras^G12C^ covalent engagers, ARS1620.^23^ Using a high-throughput cell sorting assay, relying on a GFP-K-Ras reporter construct, Zeng et al.^24^ identified potent degraders of the GFP fusion protein. However, these PROTACs failed to degrade endogenous K-Ras^G12C^. In an attempt to prevent such spurious degradation events resulting from ubiquitination of the reporter, Lin et al. recently developed a lysine-less HiBiT and NanoLuc systems^25^ that should enable better screening with fewer false positives.

**Figure 2:**
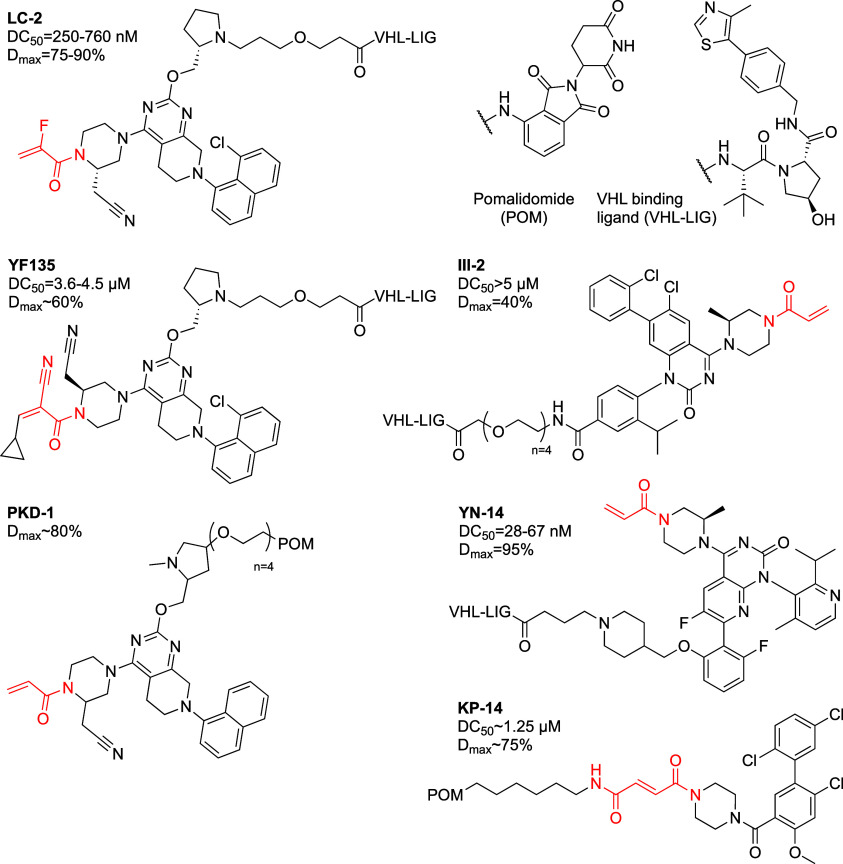
Covalent K-Ras^G12C^ PROTACs.

Several groups have developed PROTACs for K-Ras^G12C^ based on a variety of scaffolds such as sotorasib^26^ (AMG510) and adagrasib^27^ (MRTX849; Table 1a). In addition to screening different positions and chemistries for the linkers, as well as E3 ligase recruiters, the groups screened different electrophiles such as the parent acrylamides, cyanoacrylamides and fumaramide (Figure 2). The most potent PROTAC, derived from sotorasib, was YN14 reported by Yang et al.,^28^ demonstrating DC_50_ = 28–67 nM and *D*_max_ = 95%. It showed EC_50_ = 45–90 nM in viability assays (compared to 12–18 nM for sotorasib). Interestingly, the authors showed the contribution of degradation to the cell viability effects, a nondegrading analogue, showed 6-fold lower EC_50_ compared to YN-14. YN-14 was also active at 10 and 30 mg/kg in a mouse xenograft model, and showed K-Ras degradation in harvested tumors.

**Table 1:** Covalent PROTACs against Common Targets

name	ref	DC_50_	*D*_max_	cell-line	comments
(A) K-Ras^G12C^					
LC-2	(56)	590 nM	80%	NCI-H2030	
LC-2	(56)	320 nM	75%	MIA PaCa-2	
LC-2	(56)	760 nM	90%	SW1573	
YF-135	(57)	3.6 μM	60%	H358	
YF-135	(57)	4.5 μM	60%	H23	
PKD-1	(58)		80%	MIA PaCa-2	slow kinetics (72 h)
III-2	(59)	>5 μM	40%	H358	slow kinetics (2 h)
KP-14	(60)	∼1.25 μM	75%	H358	slow kinetics (48 h)
YN14	(28)	67 nM	95%	H358	
YN14	(28)	28 nM	95%	MIA PaCa-2	
(B) BTK					
**7**	(34)	136 nM	88%	K562	
RC-1	(36)	6.6 nM	75%	MOLM-14	
RC-3	(35)	6 nM	85%	Mino	
BCCov	(42)	57 nM	75%	HEK293 expressing BTK	
(C) EGFR					
PROTAC 4	(61)	216 nM	79%	H1975 (EGFR^L858R/T790M^)	
**14o**	(62)	6 nM	>95%	H1975 (EGFR^L858R/T790M^)	
SIAIS126	(63)	<30 nM	95–100%	H1975 (EGFR^L858R/T790M^)	did not outperform canertinib
CP17	(47)	1.5 nM	86%	H1975 (EGFR^L858R/T790M^)	
CP17	(47)	0.5 nM	82%	HCC827 (EGFR^del19^)	
**16c**	(64)	∼300 nM	∼68%	PC9	showed *in vivo* activity
**13**	(65)	3.5 nM	91%	HCC827 (EGFR^del19^)	
II-4	(66)	30 nM	99%	HCC827 (EGFR^del19^)	
II-4	(66)	380 nM	93%	H1975 (EGFR^L858R/T790M^)	no activity in C797S line
(D) SARS-CoV-2 M^pro^					
HP211206	(53)	620 nM		HEK293 OE M^pro^	biochemical IC_50_ = 181 nM
MPD2	(51)	296 nM		293T stably transfected with M^pro^-GFP	biochemical IC_50_ = 41 nM
					antiviral EC_50_ = 492 nM
P2	(55)	1.5 μM	90%	HEK293 cells stably transfected with M^pro^	biochemical IC_50_ = 748 nM
					antiviral EC_50_ = 0.71 μM
PROTAC 1	(52)	>80 μM		HeLa, stable HA-tagged M^pro^	biochemical IC_50_ = 21 μM

Other PROTACs exhibited lower potencies, some in the μM range. In some cases, low potency could be attributed to attenuated binding to K-Ras^G12C^, while in others it is most likely the result of poor cell permeability or impaired ternary complex formation.

We should note that only recently a noncovalent PROTAC targeting K-Ras^G12D^ was reported^29^ based on MRTX1133^30^ which showed rapid (<4 h) and potent (DC_50_ in different cell lines of 7–80 nM) degradation of K-Ras^G12D^. This potent degradation could in principle be attributed to its noncovalent nature, but it might also derive from the exceeding potency (sub-nM by SPR^30^) of MRTX1133.

##### 2.1.1.2 BTK

Several potent noncovalent PROTACs have been reported for Bruton’s tyrosine kinase (BTK)^31^ including some that have entered clinical trials.^32^ Some of these PROTACs allow to target cancer resistance mutations such as C481S which is a common resistance mutation to approved covalent BTK drugs. However, due to ample covalent targeted inhibitors, BTK also served as a testbed for the development of several types of covalent PROTACs.

**Figure 3:**
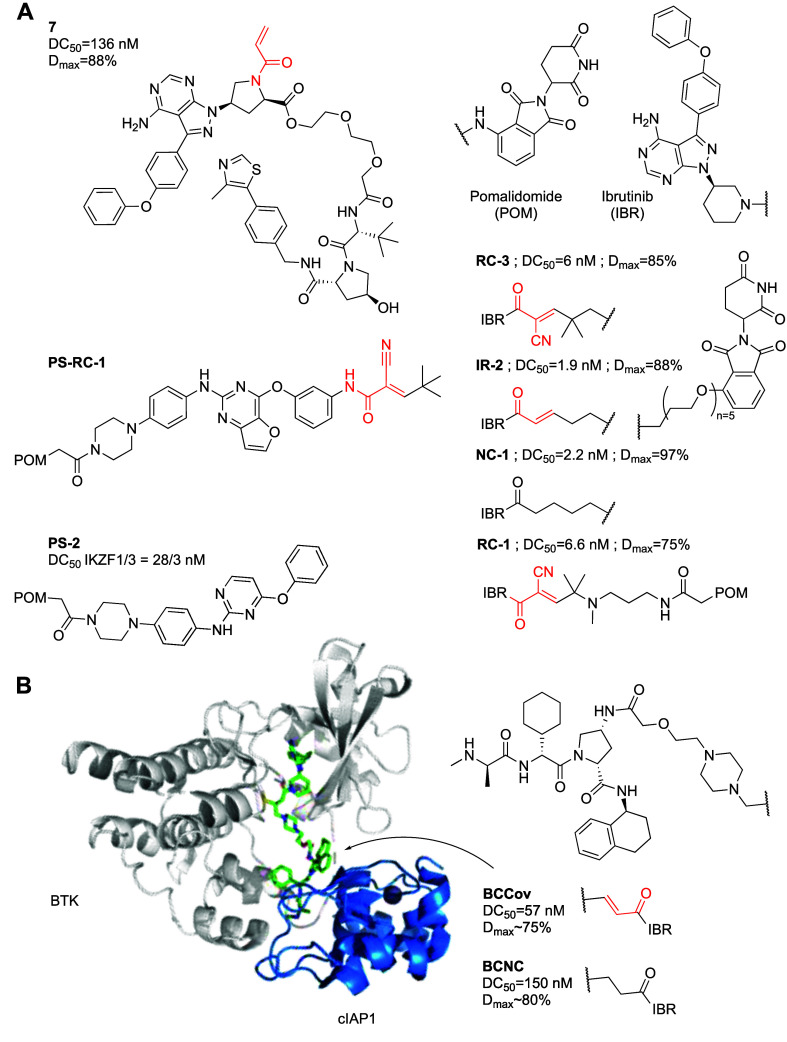
Covalent BTK PROTACs. (A) Chemical structures of various covalent BTK PROTACs. (B) Co-crystal structure (PDB 8DSO) of BTK (white) in ternary complex with covalent PROTAC BCCov (green) and E3 ligase cIAP1 (blue).

Tinworth et al.^33^ directly compared acrylamide PROTACs for BTK (based on ibrutinib) with their reduced (noncovalent) version. While the acrylamide version of the PROTAC, supposedly binding irreversibly, showed no degradation of BTK, the reduced noncovalent form exhibited potent degradation (DC_50_ = 200 nM). Nevertheless, as reviewed in this section, there are certainly examples of potent irreversible covalent PROTACs both for BTK as well as for other targets (Figure 3A; Table 1B).

Xue et al.^34^ reported acrylamide-based PROTACs such as **7** that degrade BTK (DC_50_ = 136 nM; *D*_max_ = 88%) as well as the off-target BLK (DC_50_ = 220 nM; *D*_max_ = 75%). In this case the covalent PROTACs were equal or better degraders compared to their noncovalent counterparts and engaged BTK covalently in the cell.

Both our group^35^ and the Wang group^36^ simultaneously attempted to develop reversible covalent PROTACs against BTK, using cyanoacrylamide electrophiles.^37,38^ Guo et al.^36^ synthesized RC-1, a cyanoacrylamide PROTAC based on ibrutinib that exhibited potent BTK degradation in cell culture (DC_50_ = 6.6 nM) and in mice with favorable pharmacological properties. When compared to its noncovalent and irreversible covalent (acrylamide) analogues, RC-1 exhibited both more potent degradation as well as higher selectivity. NanoBRET experiments^39^ to assess BTK and CRBN engagement in the cell suggested that improved permeability contributed to RC-1 cellular potency. It should be noted that RC-1 degraded the C481S BTK mutant with similar potency to wild-type BTK and potently degraded CSK, a noncovalent off-target of ibrutinib, which together may suggest that RC-1 degrades BTK primarily via noncovalent binding. The authors also reported reversible covalent PROTACs for the kinase FLT3.

In parallel, we reported^35^ noncovalent (NC-1), cyanoacrylamide-based (RC-3), and acrylamide-based (IR-2) PROTACs for BTK, all of which with DC_50_ < 10 nM and similar cellular permeabilities. We noticed the acrylamide IR-2 inhibited as well as degraded the mutant BTK C481S with similar potency to wild-type BTK as well as demonstrated degradation of noncovalent off-targets of BTK such as CSK and LYN, suggesting degradation is mediated primarily through noncovalent binding of the PROTAC under cellular conditions. Whereas, the cyanoacrylamide RC-3 displayed much higher potency toward WT BTK than the cysteine mutant and showed selectivity over noncovalent off-targets, suggesting it recruits BTK for degradation exclusively through covalent binding. Together these examples highlight that understanding how covalent binding affects degradation by each PROTAC requires careful measurement of the rate of covalent bond formation and dissociation. We should note that the covalent PROTACs reported by both Guo et al. and our group had comparable nM potency to published highly potent BTK PROTAC based on reversible binders.^31^

In another attempt to develop reversible covalent BTK PROTACs, Yu et al.^40^ made cyanoacrylamide derivatives of the BTK inhibitor poseltinib.^41^ These resulted in a significant loss of activity toward BTK, but surprisingly, one of them, PS-RC-1, showed very potent (10 nM) toxicity against Mino cells. They found that it is a potent degrader of IKZF3 (DC_50_ = 44 nM) and to a lower degree IKZF1 (DC_50_ = 800 nM). Systematic truncation led to PS-2, which showed improved IKZF1/3 degradation (DC_50_ = 28/3 nM respectively). Surprisingly, it also degrades BTK and CSK despite lacking the electrophile.

A group from Pfizer, went to great lengths to show that the irreversibly bound form of BTK could be degraded.^42^ They used an irreversible (BCCov) and corresponding reduced version (BCNC) BTK PROTACs, previously reported.^33^ They set up a cellular system which lacks endogenous BTK and cIAP, and followed the degradation of induced BTK after separate induction of cIAP. Both PROTACs were potent against both WT and mutant BTK. They demonstrated BCCov reached >90% irreversible labeling in cells before inducing cIAP1 expression, and could then still reach a *D*_max_ of ∼70%. Similarly, they demonstrated that purified BTK, irreversibly modified with the PROTAC, could be ubiquitinated *in vitro* and degraded in lysates. Finally, they were able to determine the first crystal structure of a ternary complex between a covalent PROTAC, its target and recruited ligase (PDB 8DSO; Figure 3B).

##### 2.1.1.3 EGFR

**Figure 4:**
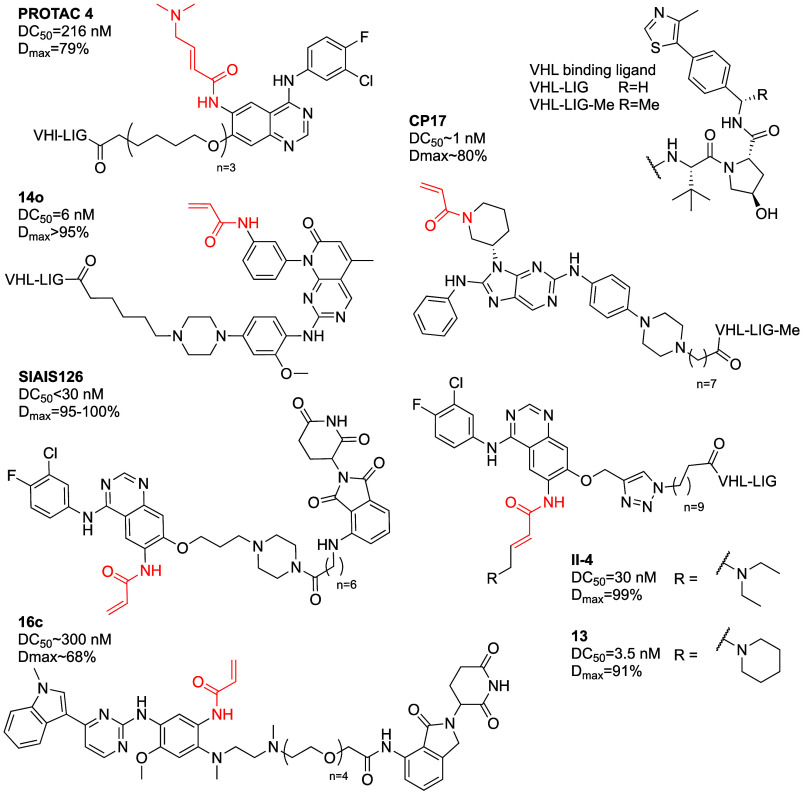
Covalent EGFR PROTACs.

Many groups designed PROTACs for EGFR based on existing covalent inhibitors such as afatinib,^43^ osimertinib,^44^ canertinib^45^ and dacomitinib,^46^ some reaching DC_50_ values in the nM range (Figure 4; Table 1C). In many cases the contribution of the covalent binding to the activity was not characterized. However, Zhao et al.^47,48^ prepared acrylamide PROTACs derived from a noncovalent EGFR inhibitor that potently inhibited cell growth of two EGFR mutant cell lines. The most potent PROTAC (CP17; Figure 4) demonstrated DC_50_ ∼ 1 nM in both cell lines with a *D*_max_ of slightly over 80%. Reduction of the acrylamide abrogated the activity only against the H1975 cell line, and the PROTAC was inactive against cells harboring the C797S mutation, suggesting a covalent mechanism. However, when they replaced the VHL recruiter with its inactive enantiomer, the compounds were no longer able to degrade EGFR, but maintained their cellular potency, suggesting that in this case it was EGFR inhibition rather than degradation that drove the cellular effect.

##### 2.1.1.4 SARS-CoV-2 M^pro^

**Figure 5:**
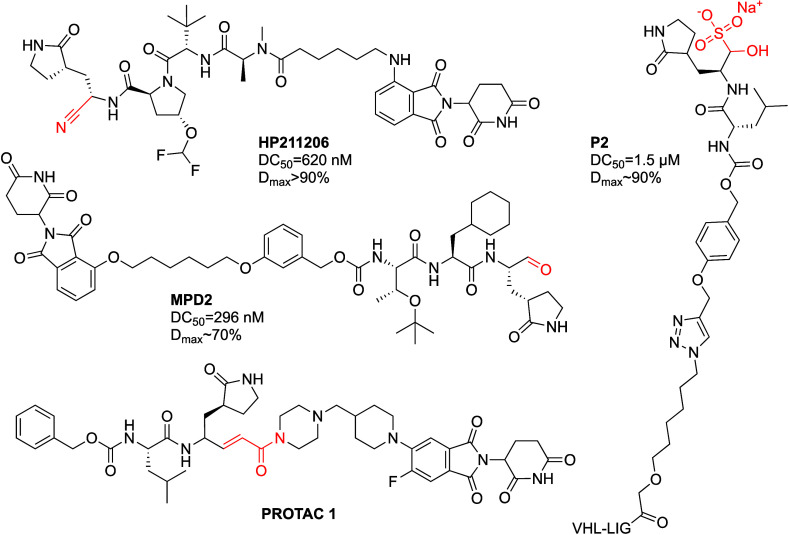
Covalent SARS-CoV-2 M^pro^ PROTACs.

Several groups attempted to design PROTACs for SARS-CoV-2 M^pro^ derived from various covalent inhibitors such as PF-073212332^49^ and GC376^50^ (Figure 5; Table 1D). This is a more challenging system for PROTACs compared to BTK, EGFR or K-Ras, since the expression of M^pro^ during viral infection is transient, and the PROTAC must degrade the expressed M^pro^ rapidly to inhibit viral replication. M^pro^ PROTACs are usually tested using cell lines that overexpress tagged or untagged M^pro^, in which the levels of M^pro^ are high and the kinetics of degradation may not be relevant for antiviral activity. Accordingly, several PROTACs that degraded M^pro^ were either inactive in antiviral assays or showed no improvement over the parent inhibitor.^51,52^

Sang et al.^53^ used a nitrile based reversible covalent inhibitor of SARS-CoV-2 M^pro^ as the basis for a PROTAC targeting M^pro^. The resulting PROTAC, HP211206, showed similar biochemical M^pro^ inhibition (IC_50_ = 181 nM compared to 151 nM for the parent inhibitor), and was able to degrade M^pro^ in HEK293 cells transfected with an M^pro^ plasmid with DC_50_ = 620 nM (48 h). However, no antiviral activity was reported for the PROTAC. Alugubelli et al.^51^ used a similar approach based on an aldehyde peptidic reversible covalent inhibitor of M^pro^, arriving at the PROTAC MPD2. It maintained an IC_50_ = 41 nM in a biochemical M^pro^ inhibition assay, and showed a DC_50_ = 296 nM (48 h) in 293T cells stably transfected with an M^pro^-GFP construct. MPD2 showed an EC_50_ = 496 nM in ACE2-A549 cells infected with SARS-CoV-2, and demonstrated M^pro^ degradation in the infected cells. It should be noted that this EC_50_ is, however, slightly weaker than that reported for the parent aldehyde.^54^

In one notable example, Cheng et al.^55^ designed a PROTAC (P2) based on GC376,^50^ which exhibited comparable antiviral efficacy to the parent inhibitor (EC_50_= 0.61/0.71 μM for GC376/P2 respectively) but rather low potency degradation (DC_50_ = 1.5 μM), suggesting that inhibition of M^pro^ is the major contribution to antiviral efficacy. The authors did show ternary complex formation between M^pro^, the PROTAC and VHL, and managed to crystallize Mpro with the PROTAC (PDB 8YLS), but detected density only for GC376, indicating weak interactions between the linker and the protein. Grifagni et al.^52^ were also able to determine a cocrystal structure of Mpro in complex with PROTAC 1 (Figure 5; PDB 8OKC) in which the covalent bond to the active site cysteine is observed, but again, there is no density for the linker and CRBN recruiter.

##### 2.1.1.5 HaloPROTACs

**Figure 6:**
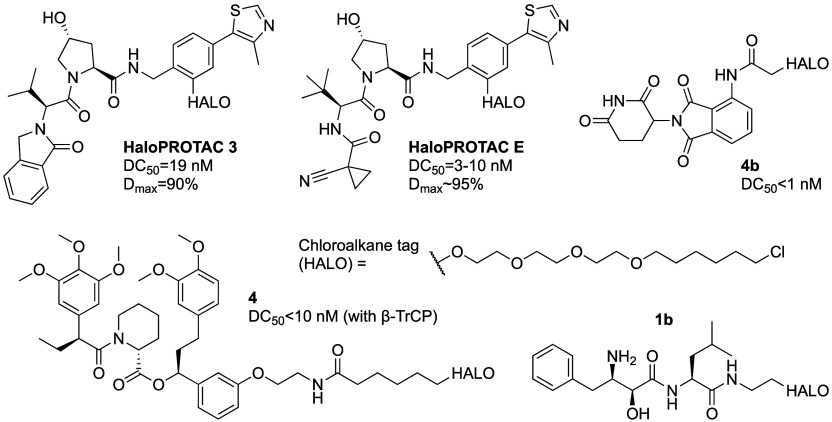
Chemical structures of HaloPROTACs

PROTACs that utilize a chloroalkane moiety to recruit a HaloTagged target protein were proven potent degraders and versatile chemical biology tools. Buckley et al.^67^ reported HaloPROTAC 3 (Figure 6) with DC_50_ = 19 nM and *D*_max_ = 90% against stably expressed GFP-HaloTag7, much better than previously reported Halo-hydrophobic tagging degrader HyT36^68^ (DC_50_ = 134 nM; *D*_max_ = 56%). The degradation kinetics of HaloPROTAC3 were fast with 50% degradation reached between 4 and 8 h. To demonstrate generality, they showed robust degradation of HaloTag7-ERK1 and HaloTag7-MEK1. HaloPROTACs were successfully used to investigate several proteins including YTHDC1,^69^ INTS11,^70^ OGT,^71^ β-catenin,^72^ and more.

Tovelll et al.^73^ optimized VHL HaloPROTACs further to HaloPROTAC E (Figure 6) and combined it with CRISPR/Cas9 endogenous protein tagging of the HaloTag to degrade two endosomal proteins SGK3 and VPS34, with DC_50_s = 3–10 nM. Its kinetics were rapid with 50% degradation within 0.5 h while reaching *D*_max_ ∼ 95% at 48 h.

Tomoshige et al.^74^ reported a similar HaloPROTAC approach using cIAP1 recruiters. HaloPROTAC 1b (Figure 6) was able to degrade HaloTag-CREB1 in the nucleus at single digit μM concentration. Ody et al.^75^ attempted to make CRBN recruiting HaloPROTACs. They tested eight compounds against HEK293 cells transiently transfected with EGFP-HaloTag2. 4b (Figure 6) degraded more than 80% at 10 nM (estimated DC_50_ < 1 nM) HaloPROTAC 3 in their hands showed DC_50_ = 3.4 nM. To show generality, they expressed Aurora B kinase, either tagged with EGFP-HaloTag2 or just HaloTag2; **4b** had a DC_50_ ∼ 10 nM for the former, and ∼100 nM for the latter.

HaloPROTACs were also used in the other direction, to identify E3 ligases that are suitable for targeted degradation but have no known ligands. In this case the ligase is expressed with an HaloTag, and the PROTAC is based on a binder to a known target. Ottis et al.^76^ tested HaloPROTAC **4** (Figure 6) against six E3 ligase-HaloTag constructs: CHIP, MARCH5, NEDD4L, parkin, SIAH1 and β-TrCP, for degradation of GFP-FKBP. All ligases except CHIP were able to induce degradation through the HaloPROTAC, but β-TrCP was especially potent with full degradation at 10 nM and some degradation even at 1 nM. β-TrCP and parkin were also demonstrated to be able to degrade endogenous kinases through appropriate HaloPROTACs.

TRAFTACs^77^ are a variation of HaloPROTACs, in which the HaloTag is installed on dCas9, which through a chimeric RNA/DNA oligonucleotide is in proximity to a target transcription factor, a HaloPROTAC is then used to recruit VHL for the degradation of the transcription target of interest. It is interesting to note that the HaloPROTAC used for this approach had to be optimized such that they would not induce the degradation of the tagged-dCas9, but rather the TF. To do so, the linker lengths to the HaloTag binding motif were significantly increased.

##### 2.1.1.6 Covalent Ligand Directed PROTACs

**Figure 7:**
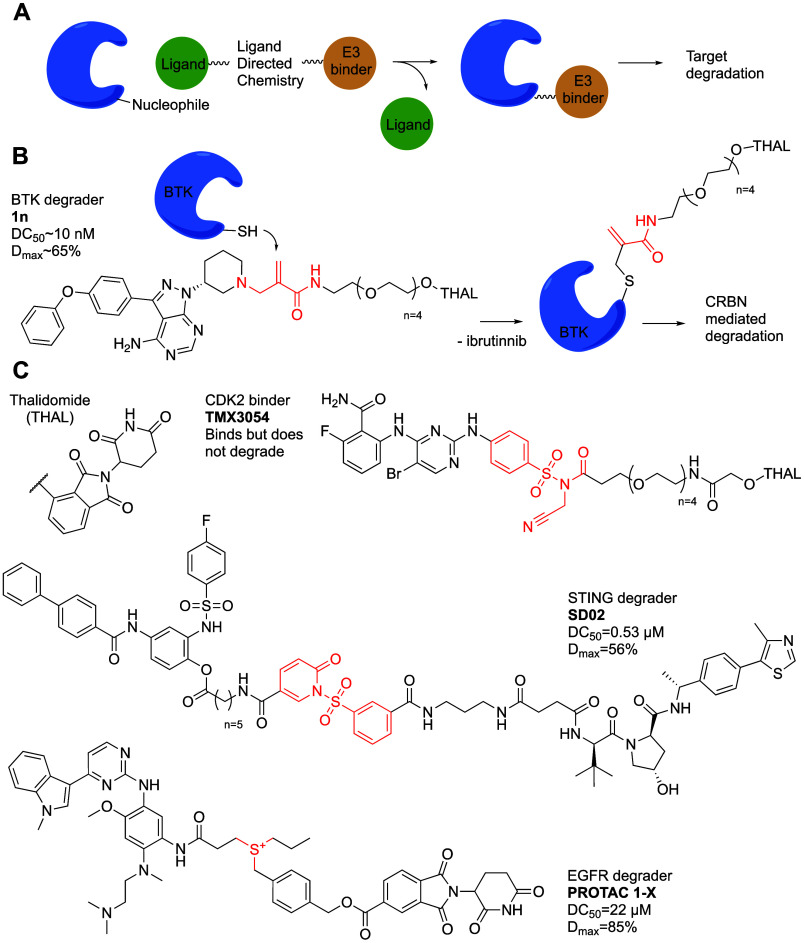
Covalent ligand directed PROTACs. (A) General scheme for covalent ligand directed PROTACs. After labeling the target protein with a degradation label, the guiding ligand acts as a leaving group and vacates the protein. (B) Chemical structure of CoLDR based BTK PROTAC **1n** with a schematic mechanism of action. (C) Chemical structures of covalent ligand directed PROTACs, the reactive centers are indicated in red.

A relatively new way to induce targeted degradation via covalent recruiters is through the use of ligand directed chemistry. We developed covalent ligand directed release chemistry^78−80^ (CoLDR) that allows the use of covalent ligands, such as ibrutinib, to irreversibly install a tag on a target protein, while releasing its guiding ligand in the process. We’ve used BTK as a model system to show that we can decorate it covalently with a CRBN recruiter^79^ (1n; Figure 7), while releasing a guiding ibrutinib molecule in the process. This resulted in potent PROTACs (DC_50_ ∼ 10 nM) however with a modest *D*_max_ ∼ 65% which might be the result of the single turnover degradation by this irreversible modality (interesting to note the *D*_max_ is similar to the irreversible BTK cIAP1 based PROTACs described above). The PROTAC was selective by global proteomics with CSK and GSPT1 the only identified off-targets that were degraded to a much lesser extent.

Teng et al.^81^ used a similar approach based on ligand-directed *N*-acyl-*N*-alkylsulfonamide (NASA) chemistry.^82^ They derivatized a CDK2 ligand with a NASA electrophile such that it will install a CRBN recruiter on CDK2 (TMX3054; Figure 7). This resulted in selective functionalization of Lys89 on CDK2. However, in this case it did not lead to degradation of CDK2, due to a failure to form a productive CDK2:CRBN complex.

Zigang Li’s group used two different group transfer chemistries to develop degraders in a ligand directed fashion. Luo et al.^83^ used this approach to target STING using sulfonyl pyridone transfer chemistry.^84^ They based their design on a reported STING inhibitor SN011^85^ and a VHL binder. The resulting PROTAC SD02 (Figure 7) labeled Tyr197 in trypsin digestion followed by LC/MS/MS analysis. In THP1 cells it degraded STING with DC_50_ = 0.53 μM; *D*_max_ = 56%. The degradation was proteasome, neddylation and VHL dependent. SD02 reduced the levels of pTBK1, pIRF3, and mRNA levels of IFN-β downstream to STING, however, not as potently as SN011 alone.

Using sulfonium transfer chemistry, Wang et al.^86^ attempted to degrade EGFR. They designed PROTAC 1-X (Figure 7) to transfer a CRBN binding ligand onto EGFR based on osimertinib as a covalent guiding ligand. It demonstrated EGFR degradation in A549 cells with a DC_50_ ∼ 20 μM (24 h) and *D*_max_ = 85%. The degradation was proteasome and neddylation dependent as well as dependent on the presence of the sulfonium. The authors applied similar chemistry for hydrophobic tagging^87^ of EGFR with an adamantane group which showed a DC_50_ = 26 μM and DC_max_ = 77%.

Though not yet proven, a possible advantage of ligand directed tagging for degradation is that it may render the target more amenable for degradation. Ligand binding typically stabilizes proteins,^88^ with this approach, the ligand vacates the binding site and the *apo* protein might be more prone to degradation. Further investigation into this approach on additional targets, and careful comparison of irreversible degraders in which the guiding ligand remains in the pocket, to equivalents in which it leaves may elucidate this point in the future.

##### 2.1.1.7 Additional Examples

**Figure 8:**
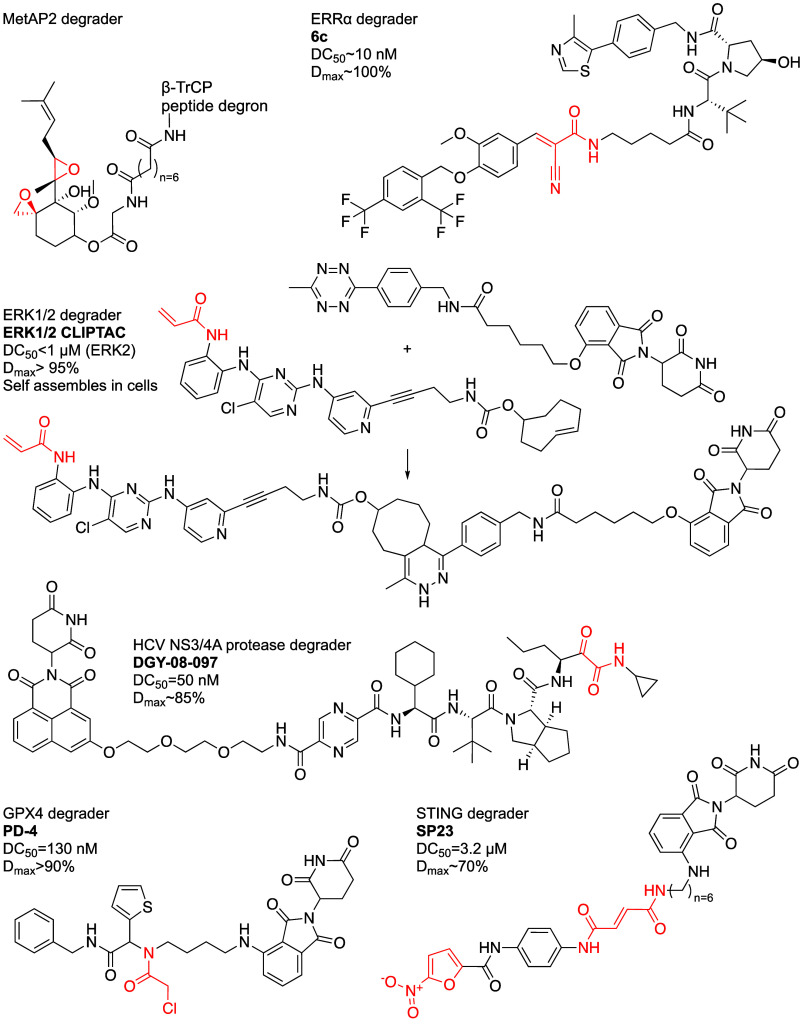
Structures of additional various covalent PROTACs.

There are several additional examples of PROTACs degrading their target based on covalent binding to said target, including the first reported proof-of-concept PROTAC^89^ that utilized the covalent natural product ovalicin to degrade MetAP-2 (Figure 8).

Lebraud et al.^90^ introduced CLIPTACs, PROTACs that spontaneously form *in situ* by copper free cycloaddition of tetrazine-labeled thalidomide and a trans-cyclooctene labeled target binder. One of their examples consisted of a covalent ERK1/2 binder, ERK-CLIPTAC (Figure 8). Treating A375 cells (1 μM; 4 h) followed by administering the CRBN recruiter (1 μM; 18 h) led to sub-μM ERK1/2 degradation. Interestingly, washing the cells after incubation with the covalent binders partially reduced ERK1/2 degradation. Indicating, degradation proceeded both through covalent and noncovalent binding to ERK1/2.

de Wispelaere et al.^91^ developed reversible covalent PROTACs for hepatitis C virus NS3/4A protease based on telaprevir, and an optimized CRBN binder. DGY-08-097 (Figure 8) shows a slightly weaker biochemical potency than telaprevir (247 nM vs 34 nM respectively), but a DC_50_ = 50 nM in an NS3/4A reporter cell line. While it was still weaker in cellular antiviral assays (EC_50_ = 750 nM vs 130 nM for telaprevir) it showed better tolerance to mutations that resisted telaprevir.

Peng et al.^92^ reported a cyanoacrylamide based PROTAC of ERRα, predicted by computational modeling to form a covalent interaction with C325. Compound **6c** (Figure 8) showed a DC_50_ slightly higher than 10 nM and *D*_max_ close to 100%. However, it did not degrade the ERRb and ERRg isoforms, which contain the same conserved cysteine, thereby casting doubt on the role of a covalent interaction in the degradation.

Liu et al.^93^ set out to develop PROTACs for STING. They based their design on a previously reported covalent binder C-170.^94^ Out of 24 tested compounds, SP23 (Figure 8) was the most potent with DC_50_ = 3.2 μM and *D*_max_ ∼ 70%, and interestingly comprised an additional fumaramide electrophile. The PROTAC was active *in vivo*, but no characterization of its covalent properties is described.

Zhu et al.^95^ used the Ugi reaction to synthesize a small series of covalent PROTACs against GPX4, based on the chloroacetamide ML-162.^96^ Of these, PD-4 (Figure 8) showed DC_50_ = 130 nM and induced lipid peroxidation, but in cellular viability assays was inferior to parent inhibitor ML-162.

To summarize this section, PROTACs that form covalent bonds with their degradation target can be potent (EGFR, K-Ras^G12C^, HaloPROTACs), selective (BTK) and address otherwise challenging targets (K-Ras^G12C^).

Reversible covalent chemistry may marry the potency and selectivity of covalent binders with the catalytic advantages of reversible PROTACs, and is relatively underexplored. As emerging and re-emerging^18^ electrophiles will be used for PROTAC design perhaps targeting additional amino acids such as lysines with (reversible covalent) salicylic aldehydes will become more prevalent.^97^ New ligand directed chemistries are also just emerging in the field of targeted degradation and may allow to expand the target scope.

With that, in developing covalent PROTACs one should also be aware of the challenges. Proteomic selectivity should be assessed for the binder, as well as the degrader. Protein synthesis rate should be taken into account, as irreversible binders would be less effective against rapidly synthesized proteins. Lastly, it is always important to characterize the contribution of the covalent bond to the degradation (by mutation, or through a noncovalent analogue) as there are many examples when in hindsight PROTACs that contain electrophiles actually degrade noncovalently.

#### 2.1.2 Covalent Binding to the Recruited Effector

E3 ligases did not evolve to bind small molecules, and therefore they are challenging for small molecule binder discovery. The binders that are in prevalent use are derivatives of E3 endogenous peptide ligands (VHL)^98,99^ or protein damage mimic (CRBN).^100^

Covalent binding was repeatedly proven to enable targeting of challenging targets and therefore was heavily used to discover new E3 ligase binders (Table 2). Phenotypic screening aided by chemoproteomics, and direct targeted screening using electrophilic libraries were especially prolific in the discovery of new covalent recruiters for targeted degradation. In addition to facilitating discovery, mathematical modeling suggests that irreversible binding to the UPS component will improve the catalytic efficiency of degradation, due to faster formation of ternary complex.^101^ Finally, such irreversible binding will increase the lifetime of degradation and maintain it following clearance of the PROTAC.

**Table 2:** Covalent PROTACs Recruiting Various Effectors to Induce Targeted Degradation

effector	target residue	name	target	DC_50_	discovery	additional targets
DCAF16	C177/C179	KB02-SLF	FKBP12	∼2 μM	chemoproteomic screen	BRD4, PARP2
RNF114	C8	XH2	BRD4	0.1–1 μM	natural product	BCR-ABL
RNF114	C8	ML2–14	BRD4^a^	14 nM	gel-ABPP screen (318)^b^	BCR-ABL
RNF4	C132/C135	CCW-28-3	BRD4	∼100 nM	gel-ABPP screen(141)	N/A
KEAP1	C151	CDDO-JQ1	BRD4	∼50 nM	natural product	N/A
KEAP1	C151	955	CDK9	9 nM	natural product	EML4-ALK
DCAF11	C443/C460/C485	21-SLF	FKBP12	1–2 μM	chemoproteomic screen	AR
DCAF11	C443/C460/C485	9	BRD2/3/4	255 nM	natural product	PDEδ, BLK
DCAF11	C443/C460/C485	HL435	BRD4	12 nM	natural product	N/A
Fem1B	C186	NJH-1-106	BRD4	250 nM	competitive-FP screen (566)	BCR-ABL
DCAF1	C1113	YT47R	FKBP12	<0.5 μM	chemoproteomic screen	BRD4
UBE2D	C111	NF90	BRD4^a^	∼100 nM	gel-ABPP screen (569)	AR
DDB1	C173	MM-02-08	BRD4^a^	∼10 nM	gel-ABPP screen (185)	AR
SKP1	C160	SJH1-51B	BRD4^a^	∼1 μM	gel-ABPP screen (1284)	AR
CRBN	H373	FS-ARV-825	BRD4	∼30 nM	covalent derivatization	N/A
VHL	S110	BRD-SF2	BRD4	17.2 μM	covalent derivatization	N/A
FBXO22	C326	UNC8732	NSD2	60 nM	BioID (proximity ligation)	XIAP
FBXO22	C326	SP3CHO	FKBP12	∼10 nM	CRISPR/Cas9 screen	XIAP
FBXO22	C227/C228	22-SLF	FKBP12	500 nM	CRISPR activator screen	BRD4, ALK

a Short isoform.

b Number indicates screened library size.

##### 2.1.2.1 Approaches for Screening, Target Identification, and Covalent Selectivity Assessment

Activity based protein profiling (ABPP) pioneered by the Cravatt and Bogyo laboratories is a powerful technique that leverages the covalent bond formed with target proteins to elucidate molecular targets, selectivity profiles and nucleophile reactivities. These have been excellently reviewed elsewhere,^102−105^ but since they are heavily used in the discovery and characterization of covalent proximity inducers, I will provide a short introduction to some of these methods.

- *Gel-Based ABPP*: Herein typically used as a method to identify covalent binders to a specific target *in vitro*. The target is incubated with potential covalent binders followed by incubation with a fluorescent reactive electrophile (typically iodoacetamide-rhodamine), and ran on a gel. Loss of fluorescent signal in the gel indicates competition for the nucleophile, and covalent binding by the query compound. The presence of multiple nucleophiles (e.g., cysteines) on the target protein may complicate this assay.
- *isoTOP ABPP*(12) (isotopic tandem orthogonal proteolysis, ABPP): This proteomic method allows to quantitatively assign a competition ratio (corresponding to% occupancy of a given cysteine) for thousands of cysteines between a query compound and a general iodoacetamide probe. An untreated cell sample and a cell sample incubated with a query compound, are then labeled with isotopically labeled iodoacetamide tags that contain a TEV cleavage site and a biotin handle. After enrichment on avidin beads, TEV is used to elute the labeled peptides that are then quantified.
- *isoDTB ABPP*(13) (isotopically labeled desthiobiotin azide tags, ABPP): This is a variation on isoTOP-ABPP that does not require the TEV cleavage for elution of the covalently labeled peptides after the enrichment step. The isotopically labeled desthiobiotin tags, that have lower affinity to the avidin beads than biotin, can be eluted by washing, circumventing the enzymatic cleavage step and simplifying the tags synthesis and size.
- *TMT ABPP*(14,15) (tandem mass tag, ABPP): Another variation on the two previous methods, where now multiple samples can be multiplexed using isotopic TMT tags, to reduce overall run time.
- *IP-MS* (immunoprecipitation followed by mass spectrometry): While not strictly restricted to covalent binders, this method proved useful in several cases to identify the targets of proximity inducers. Genetically tagging one of the interaction partners, allows following treatment, to specifically precipitate this protein with any new protein interactors induced by the treatment. Proteomics (in comparison to untreated IP) identifies potential new protein partners.

##### 2.1.2.2 DCAF16

**Figure 9:**
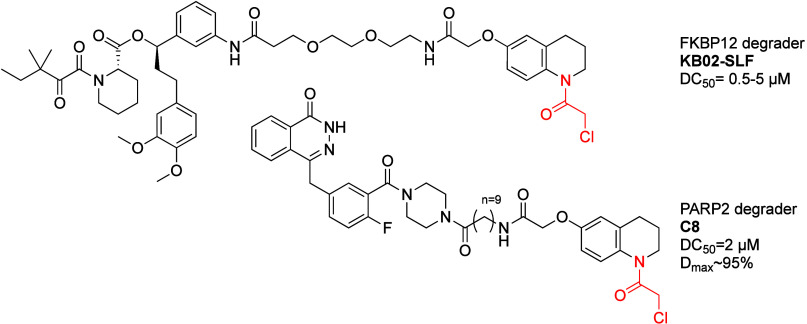
DCAF16 recruiting covalent PROTACs.

Zhang et al.^106^ set out to discover new effectors that can induce degradation by linking promiscuous covalent fragments (dubbed: “scout fragments”^107,108^) with a potent and selective ligand: SLF-targeting FKBP12.^109^ They tested only three covalent fragments in cells transfected with either FLAG-tagged FKBP12 or a nuclear localized variant. While none of the compounds affected cytosolic FKBP12, KB02-SLF (Figure 9), promoted degradation of nuclear FKBP12. This degradation was not observed in a nonreactive propanamide analogue, suggesting it is dependent on covalent binding. Washing of the compounds from cells did not affect the degradation of nuclear FKBP12.

KB02-SLF and analogues showed activity in the 0.5–5 μM range. At higher concentrations they displayed cytotoxicity, perhaps due the reactivity of the covalent scout. Both proteasome and neddylation inhibitors rescued degradation by KB02-SLF, suggesting it engages a Cullin-RING ubiquitin ligase (CRL).

To identify the ligase, the authors attempted isoTOP-ABPP,^12^ however, no cysteines on CRLs were detected with >75% engagement. They thus turned to immunoprecipitation (IP) followed by mass-spectrometry (MS) using FLAG pull-down to look for CRLs that were enriched in the presence of KB02-SLF. This identified two CRLs: DCAF16 and DTL, and more than 100 other proteins that were enriched by more than 5-fold. shRNA knockdown (KD) of either ligase showed that DCAF16 KD but not DTL rescued FKBP12 degradation by the PROTAC. CRISPR knockout (KO) of DCAF16 also rescued the degradation. Moreover, expression of DCAF16 in KO clones restored KB02-SLF-induced FKBP12 degradation.

Trypsin digestion followed by LC/MS/MS analysis of pulled-down HA-tagged DCAF16 suggested the modified cysteine is likely C177, 178, or 179. Mutagenesis studies showed that C58S-, C119S-, C173S-, and C178S-DCAF16 mutants maintained KB02-SLF-induced degradation of FKBP12 making them unlikely candidate sites of engagement for KB02-SLF. Subsequent structure determination of DCAF16^110^ suggests that C177 and C179 bind zinc and are crucial for the structural integrity of DCAF16.

To demonstrate generality, the authors next tested another PROTAC based on KB02 and JQ1 to target BRD4. KB02-JQ1 degraded BRD4 at high concentrations (DC_50_ ∼ 20 μM), and facilitated pull-down of HA-DCAF16 by FLAG-BRD4. Degradation of BRD4 was largely rescued in DCAF16 KO cells.

Competitive proteomics suggested the DCAF16 peptide 168–184 only show 10% and 40% engagement with the SLF and JQ1 based PROTACs, respectively. This data supports the notion that only fractional occupancy of the ligase is sufficient to drive target degradation.

Pu et al.^111^ used KB02 to make PROTACs targeting PARP2. They used olaparib which binds both PARP1 and PARP2 and reasoned that PARP1 has a nearby C845, that would rapidly react with the KB02 of the PROTAC, while PARP2 lacks it and will allow recruitment of DCAF16. PROTAC C8 (Figure 9) showed similar or better antiproliferative activity to olaparib across several cancer cell lines. A PROTAC based on a noncovalent version of KB02 showed 4–6 fold less activity in two cell lines in which PROTAC C8 outperformed olaparib. PROTAC C8 showed degradation of PARP2 with DC_50_ = 2 μM; *D*_max_ ∼ 95% while indeed sparing PARP1 (though the selectivity mechanism was not proven to be related to C845). Immunofluorescence experiments showed the degradation of PARP2 was limited to the nucleus. Impressively, the PROTAC outperformed olaparib in a mouse MDA-MB-231 tumor xenograft model, showing near complete tumor growth inhibition at 25 mg/kg.

##### 2.1.2.3 RNF114

**Figure 10:**
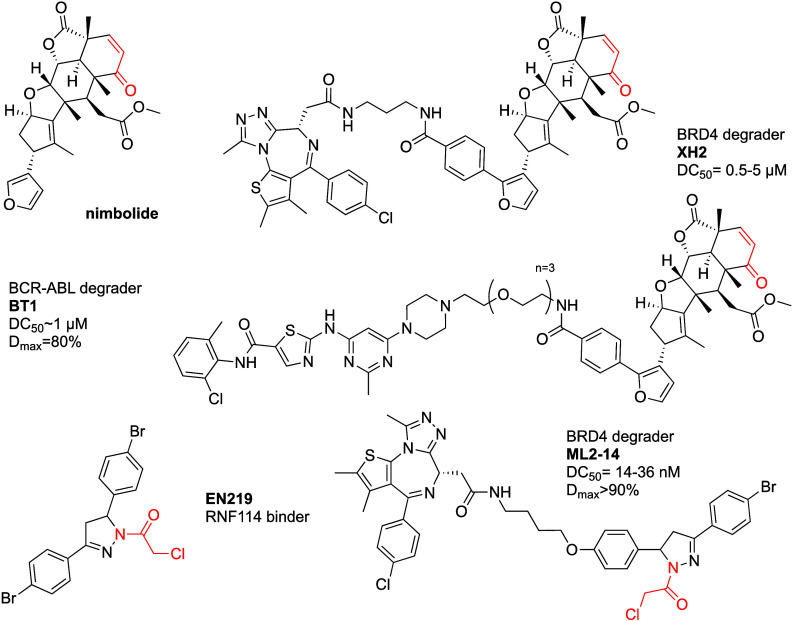
RNF114 recruiting covalent PROTACs.

Spradlin et al.^112^ set out to identify the molecular target of nimbolide (Figure 10), an electrophilic natural product with a cancer antiproliferative phenotype. They used isoTOP-ABPP in to identify the E3 ligase RNF114 as its top target. siRNA KD of RNF114 phenocopied the antiproliferative effects of nimbolide, and nimbolide’s effect was attenuated in RNF114 KD cells.

The nucleophilic site on RNF114 identified by the isoTOP-ABPP experiment was C8 which is predicted to be intrinsically disordered. An alkyne analogue of nimbolide could label purified RNF114 by in-gel fluorescence, and is competed by preincubation with nimbolide (100 μM; 30 min). Alkyne binding is also blocked by a C8A mutation. They were also able to identify the mass adduct of nimbolide on the C8 containing tryptic peptide by LC/MS/MS. Pull-down of nimbolide-alkyne labeled proteins followed by western-blot for RNF114 showed robust enrichment at 50 μM alkyne, but not at lower concentrations. Pull-down proteomics under similar conditions identified RNF114 and 114 additional proteins enriched by more than 10-fold compared to the nontreated control.

The tumor suppressor p21 was a known substrate of RNF114. The authors showed nimbolide inhibits p21 ubiquitination by RNF114. Western blot analysis and global proteomics showed p21 was stabilized by nimbolide and this effect accounted for at least part of its antiproliferative phenotype, as it could be partially rescued by knockdown of p21 and p57, another protein found to be stabilized by the proteomics.

Next the authors used nimbolide to recruit RNF114 for targeted degradation. They linked it to BRD4 ligand JQ1 resulting in XH2 (Figure 10), which retained binding to RNF114 (IC_50_ = 0.24 μM). XH2 showed BRD4 degradation at 10 and 100 nM but not at 1 μM which the authors suggest is a result of the hook effect.^113^ The degradation was sensitive to E1 Ub activating enzyme and proteasome inhibition and was rescued by RNF114 KO. Interestingly, XH2 maintained p21 stabilization while degrading BRD4, potentially dealing the cells with two orthogonal antiproliferative effects.

In follow-up work, Tong et al.^114^ reported a new extraction method that enables facile access to nimbolide from cheap commercially available health supplements. They used these large amounts of nimbolide to synthesize two PRTOACs targeting the BCR-ABL oncogene. BT1 (Figure 10) degraded BCR-ABL with DC_50_ ∼ 1 μM, and *D*_max_ = 80%. c-ABL was also degraded, but with a flat profile and *D*_max_ ∼ 50%. Neither nimbolide nor dasatinib themselves degraded BCR-ABL or c-ABL. Similar to the BRD4-nimbolide PROTAC, BT1 also led to elevated levels of p21 in K562 cells.

Looking for a more tractable synthetic handle to recruit RNF114, Lou et al.^115^ screened 318 electrophilic fragments against RNF114 (in gel-based ABPP competition with iodoacetamide-rhodamine) to identify EN219 (Figure 10) with IC_50_ = 470 nM in the gel-based assay. EN219 inhibited RNF114 autoubiquitination and p21 ubiquitination *in vitro* (50 μM; 30 min). EN219 contains a stereocenter, however the authors did not see stereospecific binding when testing the separate stereoisomers (in more recent electrophilic ligand discovery campaigns stereospecificity is considered a marker of recognition driven binding^116−118^). Trypsin digestion followed by LC/MS/MS confirmed C8 as the only site of modification.

In isoTOP-ABPP, RNF114 C8 showed a low competition ratio (∼2) compared to nimbolide which in the previous study showed a ratio of ∼5. To further assess selectivity the authors synthesized an alkyne analogue of EN219. While RNF114 was detected in a pull-down WB with the alkyne (50 μM, 90 min), it was not detected in pull-down proteomics. Competition of EN219 with the alkyne identified seven targets. The alkyne itself had 10 additional targets enriched over DMSO. Altogether these data suggest EN219 can engage RNF114 in the cell but with a concerning selectivity profile.

The authors tested three BRD4 PROTACs based on EN219. ML2–14 (Figure 10) worked best with DC_50_ of 14/36nM for the short/long isoforms of BRD4 respectively. ML2–14-mediated degradation was rescued by proteasome inhibition and E1-activating enzyme inhibition. Further, nimbolide pretreatment also rescued the degradation, suggesting it is RNF114 mediated. RNF114 KO significantly attenuated the degradation as well. Global proteomics showed, similar to the nimbolide PROTAC, both selective degradation of BRD4 (and in this case also BRD3) as well as stabilization of p21 and CTGF (also stabilized by nimbolide).

Finally, the authors show that EN219 can be harnessed to also degrade BCR-ABL, by linking it to dasatinib, it showed proteasome dependent degradation of BCL-ABL in the 0.1–5 μM range, with selectivity over c-ABL.

##### 2.1.2.4 RNF4

**Figure 11:**
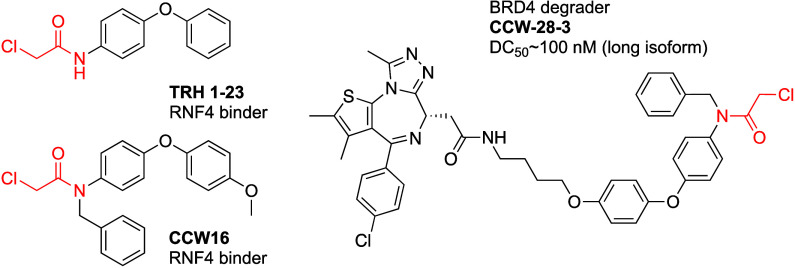
RNF4 recruiting covalent PROTAC.

Ward et al.^119^ screened 141 acrylamide and chloroacetamide fragments (50 μM) against RNF4. Of five potential hits, the authors focused on chloroacetamide hit TRH1–23 (Figure 11).

Trypsin digest LC/MS/MS studies showed that TRH1–23 (50 μM, 30 min, RT) can label two zinc coordinating cysteines C132 and C135 in RNF4, despite mutations to these cysteines previously reported to interfere with RNF4 activity, TRH1-23 treatment did not inhibit RNF4 autoubiquitination in an *in vitro* reconstituted assay. Testing 16 close analogues, identified CCW16 (Figure 11), with a lower IC_50_ value (IC_50_ = 1.8 μM). This improved binding might be due to increased reactivity which is common for choloacetamides that are based on secondary amines.^120^

The authors synthesized CCW-28-3 (Figure 11) a bifunctional linking JQ1 to CCW16. CCW 28-3 showed improved binding to RNF4 (IC_50_ = 0.54 μM). It also showed time and dose dependent degradation of BRD4 with DC_50_ ∼ 100 nM for the long isoform of BRD4. CCW28-3 mediated degradation was abrogated by pretreatment with JQ1, proteasome inhibition or E1 inhibition.

Global proteomics after CCW-28–3 treatment (1 μM, 3 h) identified BRD4 as one of the primary targets in addition to several other targets. Notably no BRD2/3 degradation was observed. Nor was RNF4 detected in the proteomics, perhaps indicating relatively low expression levels. Treatment (10 μM, 1.5 h) of cells transfected with FLAG-RNF4, showed ∼30% engagement of the protein with CCW-28–3.

To assess selectivity the authors performed an isoTOP-ABPP experiment that quantified 1114 cysteine sites, seven of which with competition ratios >4 (corresponding to >75% engagement) yet RNF4 itself was not detected. In competitive pull-down proteomics in which pretreatment with CCW16 competed with enrichment by an alkyne derivative, again seven off-targets were identified, and again RNF4 could not be detected. To validate on-target mechanism, the authors knocked out RNF4 from HeLa cells which significantly (though not completely) attenuated BRD4 degradation by CCW-28-3.

##### 2.1.2.5 Keap1

**Figure 12:**
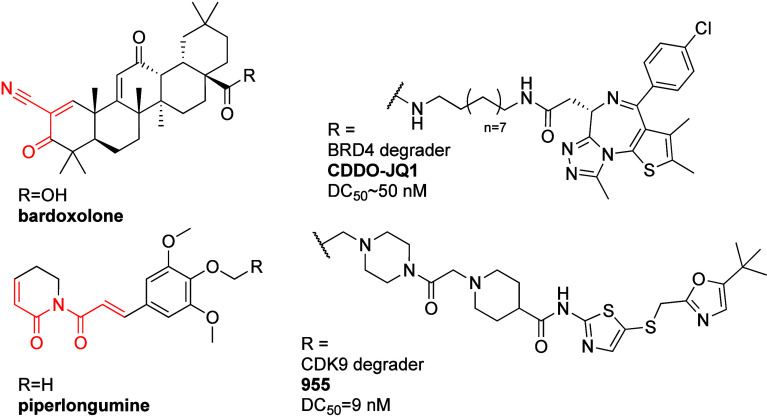
Covalent, natural product based, Keap1-recruiting PROTACs.

Tong et al.^121^ set out to recruit Keap1 as an E3 ligase for targeted degradation. To do so, they utilized the natural product bardoxolone (Figure 12) which is a reversible covalent binder of Keap1. They linked bardoxolone and JQ1 (CDDO-JQ1; Figure 12) and observed BRD4 degradation with a DC_50_ of ∼50 nM (12 h) with a potential hook-effect starting at 5 μM. At concentrations of 1 and 5 μM Keap1 levels itself were decreased by 50%.

PROTAC mediated degradation was abrogated by proteasome, E1 and neddylation inhibition. To rule out degradation due to hydrophobic tagging the authors generated three control PROTACs, two that should no longer react covalently, as well as the irreversible covalent version of bardoxolone (lacking the α-cyano group) all three control PROTACs lost their ability to degrade BRD4.

No selectivity assessments were presented neither for the effector mediating the BRD4 degradation, nor for potentially additional off-targets that might be degraded. The former as the authors note cannot be ruled out as bardoxolone is known to be promiscuous.

Relatedly, noncovalent ligands targeting the Keltch domain of Keap1 (and not the BTB domain which bardoxolone binds) were used for PROTACs.^122^ Du et al.^123^ showed that they are able to recruit Keap1 to degrade BRD4 and the kinase FAK but concluded that the target scope addressable by Keap1 recruiters is rather narrow, as “easily degradable” targets for CRBN resisted degradation by Keap1 PROTACs. Moreover, when linking the Keap1 binder to CRBN binders it was Keap1 that was degraded and not CRBN. Previously, Lu et al.^124^ reported a PROTAC for Tau using a peptide derived from Nrf2 to recruit Keap1.

Pei et al.^125^ recruited Keap1 through a second electrophilic natural product, piperlongumine (PL; Figure 12). Competitive-ABPP identified ∼300 targets for piperlongumine including nine E3 ligases. To assess its degradation capacity the authors linked it to a CDK9 inhibitor SNS-032.^126^ PROTAC 955 (Figure 12) showed DC_50_ = 9 nM for CDK9. The degradation was robust across three cell lines, and neither PL, SNS-032 nor their nonlinked combination showed degradation. Proteasome, E1, and neddylation inhibitors but not autophagy inhibitors rescued the degradation. A reduced version (of both Michael acceptors) of PL was inactive. The authors used a TurboID-CDK9 construct^127^ to identify six E3 CRLs, the only one of which that overlapped with the ABPP was Keap1. bardoxolone and dimethylfumarate could block 955-mediated degradation, as well as Keap1 KD (siRNA) or KO (CRISPR). Re-expression of Keap1 restored degradation. Finally, a nanoBRET assay in cells, showed 955 (but not its reduced counterpart) can mediate ternary complex formation at <1 μM. Global proteomics showed selectivity for CDK9 with CDK10 also being potently degraded. To demonstrate generality, the authors showed the PL linked to the ALK inhibitor ceritinib^128^ is able to degrade the fusion protein EML4-ALK at 1 μM.

##### 2.1.2.6 DCAF11

Based on their previous success^106^ with attaching scout fragments to SLF (FKBP12 ligand), Zhang et al.^129^ generated 21 chloroacetamide fragments (via the Ugi reaction^130,131^) connected to SLF via a PEG linker. These were screened (8 h; 2 μM) against four cancer cell lines that stably express cytosolic or nuclear FKBP12 fused to luciferase.

**Figure 13:**
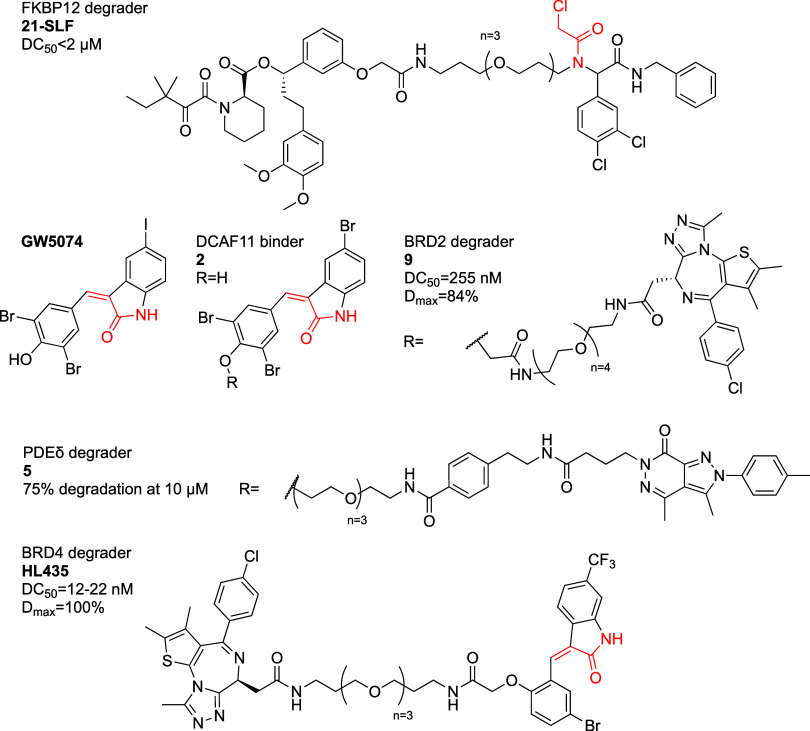
DCAF11 recruiting covalent PROTACs.

Several of the electrophilic bifunctionals exhibited activity. Interestingly it appears like the 22Rv1 cell line was more prone to induced degradation, in which 14/21 induced at least 30% nuclear degradation, compared to H2122 in which no compound but the positive control induced any type of degradation. The authors focused on 21-SLF (Figure 13). Its degradation was blocked by free SLF, proteasome and neddylation inhibition. 21-SLF also induced the polyubiquitination of cytosolic and nuclear FKBP12. The 21-SLF propanamide analogue lacking the electrophilic moiety could not induce degradation, suggesting covalent binding is necessary for activity.

To identify the potential proteins mediating the degradation the authors used co-IP of FLAG-tagged FKBP12 in cells treated with 21-SLF (10 μM, 2 h). DCAF11 was among the top enriched hits. 21-SLF degraded FKBP12 in DCAF11-WT, but not in DCAF11-KO cells. Expression of HA-tagged DCAF11 in the KO cells restored 21-SLF mediated FKBP12 degradation, and HA-tagged DCAF11 coimmunoprecipitated with FLAG-FKBP12 in the presence of 21-SLF. Global proteomics indicated selective degradation of endogenous FKBP12 in WT but not DCAF11-KO cells. The only off-target detected was GSTO1 that degraded regardless of DCAF11 expression. GSTO1 is well precedented to react with chloroacetamides^132,133^ and the authors hypothesize 21-SLF labels it and potentially destabilizes it, leading to DCAF11 independent degradation.

Having identified DCAF11 as the ligase mediating 21-SLF activity, the authors rescreened five other SLF-bifunctionals in DCAF11-WT and KO cells. Except KB02-SLF which maintained its activity in the DCAF11-KO cells, one other compound also maintained FKBP12 degradation in the KO cells, suggesting it might be engaging an alternative ligase.

To identify the cysteine target of 21-SLF, the authors expressed cysteine to alanine mutations of DCAF11 together with Luc-FKBP12 and monitored degradation by 21-SLF. only a triple C443A/C460A/C485A mutant rescued degradation completely. The DCAF11 triple mutant also failed to form a complex with FKBP12 in 21-SLF treated cells.

An isoDTB-ABPP^13^ experiment with 21-SLF (10 μM, 2 h) in HA-DCAF11-transfected cells showed limited engagement with C460 in DCAF11 and only six additional significant off-targets, including C32 of GSTO1, out of more than 12,000 identified cysteines.

Finally, to demonstrate generality of DCAF11 recruitment, the authors synthesized electrophilic PROTACs bearing the chloroacetamide moiety of 21 linked through a PEG linker to an androgen receptor (AR) ligand. It degraded AR in WT cells in a concentration dependent manner, but not in DCAF11-KO 22Rv1 cells. At 10 μM, it led to 90% depletion of AR.

The authors noted that 21-SLF only showed activity in 22Rv1 cells out of the panel of four cell lines. While DCAF11 is expressed in all four lines, it has higher expression in 22Rv1, potentially suggesting a minimum expression level of ligase expression is required for effective degradation. The authors also wonder if C460, being highly conserved across DCAF11 orthologues, might represent a functional site for DCAF11 in recognizing endogenous electrophiles, in response to, for instance, oxidative stress in cells.

Two additional groups identified DCAF11 as an effector that could drive targeted degradation, both arriving at it, at about the same time, by examining a natural product that was reported to induce lysosomal degradation.

Xue et al.^134^ set out to explore lysosomal degradation of PDEδ. To that end they used their previously developed PDEδ binders^135,136^ and GW5074 (Figure 13) that had been identified as an LC3B binder.^137^ Compound **5** (Figure 13) showed PDEδ degradation in three different cell lines, with about 75% degradation at 10 μM.

Autophagy inhibitors Chloroquine (CQ), NH_4_Cl or Bafilomycin A1 (Baf A1) could not rescue PDEδ degradation by **5**, nor could LC3B knockout, suggesting the degradation is not mediated by autophagy. In contrast proteasome inhibitors, abrogated PDEδ degradation by **5** as did neddylation inhibition, suggesting degradation by a CRL. To explore the generality of the recruiter the authors made a series of bifunctionals linking JQ1 with the arylidene-indolinone through different linkers. All bifunctional probes induced degradation of BRD2, BRD3 and BRD4 at 3 μM. Compound **9** (Figure 13) induced degradation of BRD2 with a DC_50_ = 255 nM and *D*_max_ = 84%. Interestingly, up to 50 μM concentration, almost no hook effect was observed for BRD2 and BRD4, but at 10 μM, a strong hook effect was observed for BRD3. Autophagy inhibitors did not rescue BRD2 degradation but proteasome inhibitors did. The authors made another bifunctional based on ibrutinib which showed degradation of BLK and BTK respectively in ramos cells, that was abolished by proteasome or neddylation inhibition but not by CQ.

To identify the ligase responsible for the inhibition the authors performed a CRISPR/Cas9 screen in a BRD4 reporter cell line transduced with a CRL-focused sgRNA library targeting 495 genes. DCAF11 was identified as the most significant CRL substrate receptor. To validate DCAF11 as the recruited ligase the authors used siRNA knockdown that rescued BRD2 degradation induced by **9**. Additionally, they generated stable cell lines with CRISPR knockout of DCAF11 in which, BRD2 degradation by the compounds was abolished, as well as BRD3/4 and PDEδ degradation by **5**. To confirm direct target engagement the authors expressed FLAG-tagged BRD2 and HA-tagged DCAF11 in Hep G2 cells. Co-immunoprecipitation of FLAG-BRD2 enriched DCAF11 only in the presence of compound **9**. To test if the indolinone moiety alone bound DCAF11 in cells, the authors made a biotinylated probe of it. The probe was able to pull down DCAF11, and was partially competed by the nonbiotinylated indolinone. No selectivity results were reported as to what else the indolinone might be binding in the cell.

α,β-Unsaturated indolinones contain a potential electrophilic Michael acceptor, and since DCAF11 was already demonstrated to be covalently ligandable, the authors wanted to assess covalent binding of **9** to DCAF11. First, they synthesized the reduced version, that did not induce degradation of BRD2. Then, they followed with a washout experiment. Three hours post washout of MZ1, a noncovalent BET proteins degrader BRD2 levels increased, whereas with compound **9**, BRD2 remained at low levels up to 24 h after washing. Moreover, blocking cysteines C443, C460 and C485 by treatment with the recruiter reported by Zhang et al.,^129^ attenuated the degradation of BRD2 by compound **9**, implying they engage a same cysteine target. Reconstitution of a triple C443A, C460A, and C485A mutant to DCAF11 KO cells did not restore BRD2 degradation by compound **9**, whereas reconstitution of the WT did restore degradation, further supporting one of these as the target cysteine.

The authors explored the SAR of 29 arylidene-indolinone analogues for rescuing BRD2 degradation in a competitive in-cell western assay system.^138^ Indeed, JQ1 or PDEδ ligand linked bifunctionals based on these improved binders, led to improved degradation in cells.

An indirect way to assess proteomic selectivity is to evaluate the compounds’ effect on viability. The monovalent DCAF11 ligand **2** (Figure 13), which contains the electrophilic moiety, showed GI_50_ > 30 μM in Jurkat- and Hep G2 cells, compared to the chloroacetamide DCAF11 ligand reported by Zhang et al.^129^ that showed GI_50_ = 1.7–6.5 μM. The indolinone-JQ1 based degraders displayed GI_50_ = 90–200 nM in Jurkat cells, 6- to 10-fold more potent than nonelectrophilic counterparts.

As noted, a second group, Wang et al.^139^ were also inspired by the reported lysosomal degraders^137^ to make JQ1-linked bifunctional molecules. Their best compound, HL435 (Figure 13) showed marked cellular viability effects with EC_50_ = 0.38 and 0.21 μM against MCF7 and MDA-MB-231 respectively. HL435 had a DC_50_= 12–22 nM in the two cell lines (12 h) with rapid kinetics (half-life of ∼1.3 h at 0.5 μM compound). To validate the degradation mechanism the authors attempted to rescue degradation by autophagy inhibitors bafilomycin and CQ, neither were able to stabilize BRD4 in the presence of HL435, nor did knockout of ATG5 or ATG4B suggesting the degradation is not lysosomal. E1 ubiquitin-activating enzyme inhibitor or proteasome inhibitors did rescue compound mediated degradation, as did neddylation inhibition.

To identify the effector, the authors performed a pooled CRISPR interference screen, in a BRD4_BD1_ reporter cell line, comprising sgRNAs against 993 genes from the proteasome pathway. Components of the CRL4^DCAF11^ complex, including CUL4B, RBX1, DDB1, and DCAF11, were among the top positive hits. Individual knockout of these genes using two separate sgRNA blocked the BD1 reporter degradation upon HL435 treatment, as well as endogenous BRD4. Pull-down of HA-tagged DCAF11, identified FLAG-tagged BRD4_BD1_ only in the presence of HL435, suggesting ternary complex formation.

The authors demonstrated potent toxicity of HL435 in cancer cell lines, most notably a prostate cancer cell line 22RV1, with LD_50_ = 8.7 nM (compared to 157 nM for JQ1) and showed the compound blocks the cell cycle and induces apoptosis to a higher level than JQ1. They proceeded to evaluate HL435 in a mouse xenograft tumor model using MDA-MB-231 cells. Mice were treated daily with HL435 (20 mg/kg) 6 days per week. After 27 days, the treatment group showed tumor growth reduction and tumor weight reduction of ∼50% compared to the vehicle, with no effect on the mice body weight, or any obvious toxicity.

##### 2.1.2.7 FEM1B

**Figure 14:**
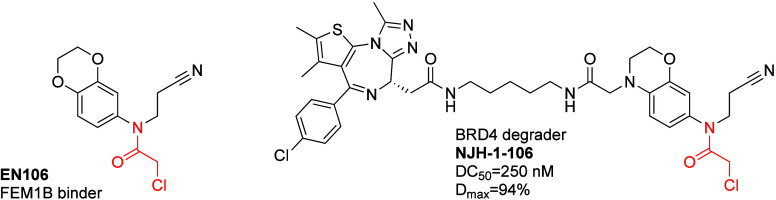
FEM1B recruiting covalent PROTAC.

To identify a covalent FEM1B binder, henning et al.^140^ screened a library of 566 covalent ligands at 100 μM in a competitive fluorescence polarization (FP) assay against a TAMRA-conjugated FEM1B substrate degron: FNIP1^562–591^ binding to recombinant mouse FEM1B. Six compounds showed >50% inhibition, of which EN106 (Figure 14) showed the most prominent inhibition with IC_50_ = 2.2 μM in the FP assay. LC/MS/MS analysis of FEM1B tryptic digest revealed an EN106 adduct only on C186. This cysteine was previously shown to be critical for FEM1B substrate recognition via a zinc mediated interaction.^141,142^ A noncovalent analogue of EN106 lost the ability to inhibit the FNIP degron binding.

To assess cellular engagement the authors synthesized an alkyne-functionalized derivative of EN106. The alkyne probe improved potency of FNIP binding inhibition (IC_50_ = 0.67 μM). The alkyne was able to pull-down FEM1B from cells. While iso-TOP ABPP with EN106 failed to identify a FEM1B derived peptide, quantitative proteomics of the alkyne pull-down identified FEM1B as one of the top-enriched proteins. As an orthogonal measure of cellular engagement, the authors assessed degradation of GFP linked to an FNIP1 degron. EN106 significantly stabilized FNIP1 degron-GFP levels in FEM1B- overexpressing cells. In cells with endogenous FEM1B levels, EN106 increased FNIP1 reporter levels only in the 20 μM dose. Stabilization of mitochondrial FNIP1 impairs mitochondrial activity, as measured by oxygen consumption rate. In accordance with stabilizing FNIP1, EN106 significantly reduced mitochondrial oxygen consumption.

To attempt targeted FEM1B mediated degradation, the authors linked EN106 to JQ1, via six different linkers. These bifunctional compounds all displayed some extent of BRD4 degradation. NJH-1-106 (Figure 14) showed the best activity with a DC_50_ of 250 nM and *D*_max_ = 94% for BRD4. NJH-1-106 maintained competitive binding to FEM1B with IC_50_ = 1.5 μM in a competitive FP against the FNIP1 degron. Its BRD4 degradation was time-dependent and it displayed activity in three different cell lines.

A noncovalent version of NJH-1-106, did not inhibit FEM1B binding to FNIP1 degron and did not degrade BRD4. Degradation could also be rescued by proteasome and neddylation inhibition, as well as by preincubation with EN106 or JQ1. FEM1B knockout attenuated but did not completely rescue BRD4 degradation by the PROTAC, a fact the authors attribute to residual wild-type cells in the nonclonal FEM1B KO population or to other potential off-target E3 ligases. Global proteomics profiling of NJH-1-106 (1 μM; 12 h) identified only BRD4 and one additional off-target as down-regulated, however BRD4 was depleted by <50%. Finally, to demonstrate generality beyond BRD4, the authors linked EN106 to dasatinib, and demonstrated degradation of BCR-ABL and c-ABL in K562 cells.

##### 2.1.2.8 DCAF1

**Figure 15:**
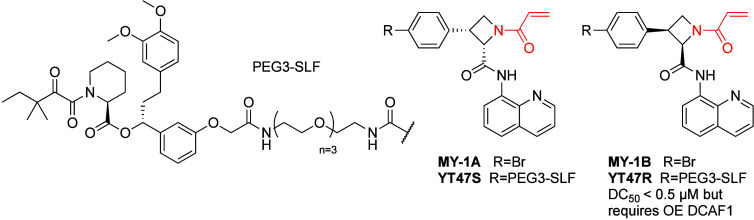
DCAF1 recruiting stereoselective covalent PROTAC.

Tao et al.^117^ profiled four azetidine-acrylamide stereoprobes (all four possible diastereomers) using cysteine-directed MS-ABPP^107,108,143^ in primary human T-cells. Altogether >10,000 cysteines were quantified, of which they focused on C1113 of the E3 ligase adaptor DCAF1. This cysteine was engaged stereoselectively by probe MY-1B (>90%; Figure 15). Other quantified cysteines in DCAF1 were unaffected by MY-1B. An alkyne derivative of MY-1B (but not of MY-1A; Figure 15) was able to label WT-DCAF1 at concentrations as low as 12.5 μM (1 h), but not a C1113A mutant, when these recombinant proteins were spiked into HEK293T lysates. MY-1B pretreatment (2 h; but not MY-1A) was able to block the alkyne engagement at concentrations of 25 μM and above.

Based on these results the authors continued to functionalize MY-1B into a PROTAC by linking it to SLF (YT47R; Figure 15). They also generated the corresponding negative control based on the inactive enantiomer (YT47S; Figure 15). This bifunctional molecule (but not the negative control) was able to block MY-1B binding to recombinant DCAF1, and was also able to cause a dose-dependent shift in the protein band as assessed by coomassie staining, likely due to their >1000 Da MW. To assess their degradation activity, the authors cotransfected HEK293T cells with FLAG-tagged DCAF1 and HA-tagged FKBP12, followed by 24 h compound treatment.

Degradation of HA-FKBP12 was observed for the PROTAC in cotransfected cells but not in cells transfected only with HA-FKBP12, suggesting endogenous DCAF1 levels may not suffice to support degradation. The enantiomeric control did not degrade FKBP12, nor was it degraded with the PROTAC in cells transfected with a DCAF1 C1113A mutant. YT47R-mediated degradation of HA-FKBP12 was blocked by pretreatment with proteasome or neddylation inhibitor as well as by SLF or MY-1B. Global proteomics in cotransfected cells after treatment with YT47R showed FKBP12 as one of the most statistically significant degraded proteins, although the degradation magnitude was modest.

To assess ternary complex formation, cotransfected cells were treated with YT47R (or the control enantiomer YT47S) in the presence of MG132. Immunoprecipitation of the HA-tagged FKBP12 showed DCAF1 only in the PROTAC treated cells but not with the control enantiomer or cells transfected with C1113A DCAF1. Interestingly, increased polyubiquitination and ternary complex formation for YT47R over YT47S were also observed in cells only transfected with HA-FKBP12, mediated by endogenous DCAF1. While insufficient to induce degradation, the authors speculated that better engagers might be able to support degradation, and point to the fact that YT47R only engaged ∼20% of C1113 (at 5 μM).

To demonstrate generality the authors synthesized two bifunctional probes connecting MY-1B or the control MY-1A to JQ1 through a PEG3 linker. In-gel competition with the alkyne probe, showed the active PROTAC retained binding to DCAF1 and degraded BRD4 with DC_50_ ∼ 200 nM in cells transfected with FLAG-DCAF1, in a stereoselective and C1113 dependent manner. Degradation, again required exogenous expression of DCAF1.

##### 2.1.2.9 UBE2D

**Figure 16:**
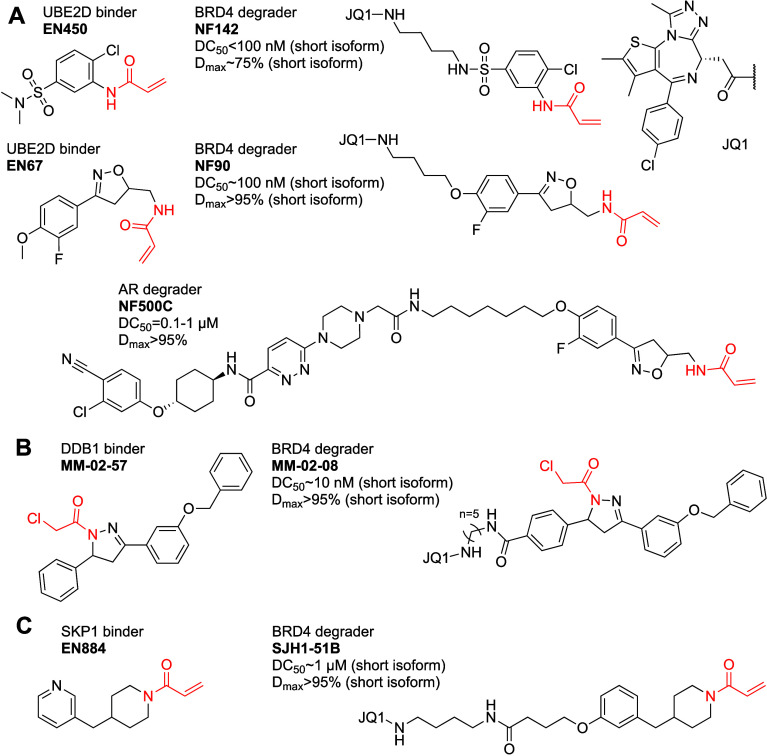
Covalent recruiters and PROTACs for non-E3 ligase UPS components. (A) UBE2D, (B) DDB1, (C) SKP1.

Based on their previously discovered phenotypic hit EN450 (Figure 16A; see section 3.5) which targets UBE2D C111, Forte et al.^144^ made a bifunctional molecule linking EN450 to JQ1. This PROTAC was able to show ∼70% degradation of the short isoform of BRD4 at concentrations of 0.1–10 μM, with no effect on the long isoform.

To find an improved recruiter, the authors screened 569 covalent fragments by gel-based fluorescence competition with an iodoacetamide probe. The top hit EN67 (Figure 16A) was able to completely outcompete the IA probe at 10 μM. Through *in vitro* ubiquitination assays, the authors showed that EN67 does not block the intrinsic activity of UBE2D, furthermore, tryptic digestion followed by LC/MS/MS demonstrated selective targeting of C111 without binding to the catalytic C85. To assess target engagement the authors synthesized an alkyne derivative of EN67; a pull-down Western blot using this alkyne (50 μM, 24 h) showed engagement with UBE2D. A competitive iso-DTB experiment against EN67 (50 μM, 4 h) identified ∼3800 cysteine sites, with UBE2D showing a modest competition ratio of ∼1.3 (corresponding to ∼25% occupancy). Numerous other peptides showed higher competition ratios but with lower significance values.

To attempt to recruit UBE2D for targeted degradation the authors synthesized five bifunctionals linking EN67 to JQ1. Of the five, only one linker (NF90; Figure 16A) enabled degradation of the short isoform of BRD4 (with no effect on the long isoform). NF90 was not only selective for the short isoform of BRD4 but also did not degrade BRD2/3. NF90 mediated degradation could be rescued with proteasome and neddylation inhibitors, which suggests that UBE2D is not sufficient to drive degradation of targeted proteins alone. Knockdown of the four UBE2D isoforms attenuated the degradation (however, such knockdown may affect many effectors of proteasomal degradation). Surprisingly, a nonreactive reduced version of NF90, was still able to compete with IA for binding to UBE2D and to induce BRD4 short isoform degradation (10 μM; 24 h) perhaps suggesting strong reversible recognition of the target (although that might not explain the competition results with IA).

To demonstrate generality, The authors linked EN67 to the AR-targeting ligand from the PROTAC ARV-110.^145^ NF500C (Figure 16A) showed near complete depletion of AR at 1 μM (24 h). Global proteomic profiling of NF500C (10 μM; 24 h) showed marked selectivity for AR. A nonreactive analogue of NF500C, with the acrylamide reduced, still degraded AR to an equivalent degree as NF500C (10 μM, 24 h).

##### 2.1.2.10 DDB1

Meyers et al.^146^ screened 185 covalent fragments (50 μM, 30 min) by Gel-based ABPP, against the DDB1 core adaptor protein of CRLs. Their top hit was previously identified as a hit against RNF114^115^ therefore they tested five chloroacetamide pyrazoline analogues and identified MM-02-57 (Figure 16B) which was able to label DDB1 but not RNF114.

An alkyne derivative of MM-02-57 maintained similar binding to purified DDB1. Trypsin digest LC/MS/MS analysis identified C173 as the target site on DDB1. MM-02-57 (50 μM, 30 min) did not inhibit Thalidomide induced ubiquitination in an *in vitro* assay, suggesting it may be suitable as a handle for targeted degradation. A pull-down WB using the alkyne probe showed it is able to pull-down DDB1 from cells (although only partially) and this pull-down can be competed by MM-02-57, suggesting target engagement in cells.

In an isoDTB-ABPP experiment to assess the proteomic selectivity of MM-02–57, out of 8974 cysteines quantified, 24 targets showed competition ratios >8 including DDB1 C173 that showed a ratio of 8.4, indicating 88% engagement of DDB1. Numerous additional cysteines showed competition ratios >4. The more prominent off-targets included the E3 ligases KEAP1 and ZNRF2.

The authors made six PROTACs linking MM-02-57 onto JQ1. While all versions degraded BRD4 (to different extent) they all only degraded the short isoform with no effect on the long isoform. The most potent degrader was MM-02-08 (Figure 16B) with DC_50_ ∼ 10 nM. MM-02-08 did not impair cell viability after 24 h of treatment. BRD4 Degradation was rescued by pretreatment with proteasome or neddylation inhibitors, as well as by DDB1 knockdown. When tested in a different cell line (MDA-MB-231) both short and long isoforms of BRD4 were degraded, perhaps indicating a sort of isoform regulation rather than degradation selectivity. In an *in vitro* reconstituted system consisting of UBE1, UBE2, CRL4, NEDD8, RBX1, and FLAG-BRD4 monoubiquitination could be detected, adding MM-02–57 showed a trace of poly ubiquitination and adding DDB1 increased the signal of the polyubiquitination.

The noncovalent analogue of MM-02-08 did not show DDB1 binding, nor BRD4 degradation. Whereas swapping the chloroacetamide electrophile for an acrylamide, maintained binding to DDB1 and degradation of BRD4 albeit weaker than MM-02-08.

To demonstrate generality the authors linked MM-02-57 to an AR-targeting moiety borrowed from the clinical AR PROTAC ARV-110.^145^ One PROTAC reached more than 50% degradation of AR in LNCaP cells (∼1 μM; 24 h).

##### 2.1.2.11 SKP1

Hong et al.^147^ aimed to identify a recruiter for SKP1. They screened 1284 covalent fragments (50 μM; 1 h; RT) via gel-based ABPP, against pure SKP1-FBXO7-CUL1-RBX1 complex. EN884 (Figure 16C), the top hit, showed dose–response displacement of IA-rhodamine (EC_50_ ∼ 10 μM).

LC/MS/MS analysis identified one modified site by EN884, C160 on SKP1. This cysteine resides at the C-terminus of SKP1 in a region predicted as intrinsically disordered. onsistent with the prediction, EN884 did not bind monomeric SKP1 alone, suggesting an additional component of the complex was required to template binding.

The authors made an alkyne derivative of EN884, that was able to pull down SKP1 from HEK293 cells. An isoDTB-ABPP experiment identified only 7% occupancy of C160 of SKP1 for EN884. A pull-down proteomics experiment with the alkyne derivative enriched SKP1, but also 414 other proteins were enriched, suggesting very poor selectivity.

Nevertheless, the authors synthesized four PROTACs linking JQ1 to a derivative of EN884. The most robust of which, SJH1–51B (Figure 16C), showed a DC_50_ ∼ 1 μM for only the short isoform of BRD4. In MDA-MB-231 cells both short and long isoforms were degraded, global proteomics showed moderate selectivity toward BRD4. The degradation was sensitive to proteasome and neddylation inhibition and to SKP1 knockdown.

Surprisingly, the reduced, noncovalent version of SJH1-51B, was still able to degrade BRD4 short isoform, albeit slower, and to displace iodoacetamide in the in-gel ABPP assay from SKP1, only in the context of the complex. This is particularly surprising due to the fragment-sized recognition element, that is unlikely to potently bind the complex reversibly.

To show generality, the authors synthesized four PROTACs incorporating an AR tarageing ligand. All four showed some level of degradation, with one of them in particular showing sub-μM AR degradation, with some selectivity toward AR in global proteomics. Here too, noncovalent analogues showed equipotent AR degradation, further suggesting that covalency is not driving degraders based on this recruiter.

##### 2.1.2.12 CRBN

**Figure 17:**
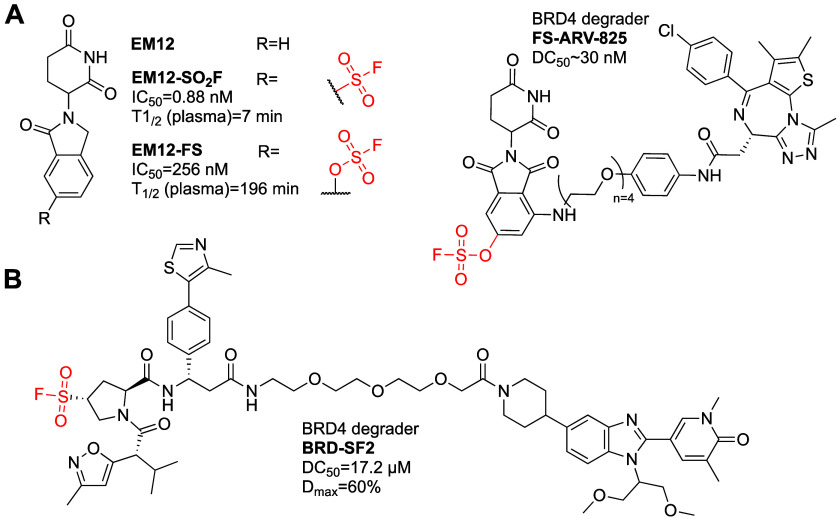
CRBN and VHL covalent recruiters. (A) Structures of covalent CRBN recruiting ligands and PROTAC. (B) Structure of covalent VHL recruiting PROTAC.

Cruite et al.^148^ installed sulfur(VI) electrophiles on the 5 and 6 positions of the CRBN binder EM12 (Figure 17A) in an attempt to turn it into a covalent binder. To assess cellular engagement, they used a NanoBRET assay that quantifies BRET signal following displacement of a fluorescent CRBN tracer from NanoLuc-tagged CRBN.^149^ A sulfonyl-fluoride at the 5 position (EM12-SO_2_F; Figure 17A) was extremely potent in this assay (IC_50_ = 0.88 nM) but also unstable (*T*_1/2_ = 7 min in human plasma). A sulfonyl fluoride at the 6 position was 80-fold less active, indicating exact placement of the electrophile contributed to potency. A fluorosulfate at the 5 position (EM12-FS; Figure 17A) gave a good balance of potency and stability with IC_50_ = 256 nM, Human plasma *T*_1/2_ = 196 min; HLM *T*_1/2_ > 145 min and human hepatocyte *T*_1/2_ > 217 min. It is interesting to note that a fluorosulfate at the 4 position, resulted in similar potency (IC_50_ = 310 nM). EM12-SO_2_F and EM12-FS were shown to irreversibly label CRBN by LC/MS exclusively at His353 (1:1 ratio, 4 h for SO_2_F, 24 h for FS; RT). In terms of neo-substrate degradation, EM12-SO_2_F did not show degradation of any neo-substrates by proteomics and in fact was able to block IKZF1 degradation by Lenalidomide. Supposedly due to imposing a new conformation on His353. EM12-FS, also did not degrade IKZF, but acquired a new neo-substrate NTAQ1 which was robustly and selectively degraded.

In a follow-up study Nowak et al. attempted to convert this covalent CRBN binder into a PROTAC.^150^ They installed the fluorosulfate at the corresponding position of AVR-825, a JQ1/CRBN based degrader of BRD4^151^ resulting in FS-ARV-825 (Figure 17A). It showed 50% labeling of CRBN *in vitro* (1:1 ratio; 24 h; RT). In the NanoBRET assay the parent ARV-825 showed an IC_50_ = 57 nM while FS-ARV-825 was 15-fold less potent with IC_50_ = 850 nM. In terms of degradation FS-ARV-825 showed a DC_50_ ∼ 30 nM (compared to ∼10 nM for the noncovalent version in this system) but retained the same DC_50_ (at 24 h) after preincubation with a potent (reversible) CRBN binder, whereas the noncovalent PROTAC activity diminished significantly with such competition. Also, as expected from an irreversible PROTAC, it was resistant to washout of the cells. In terms of selectivity, FS-ARV-825 clearly degraded BRD2/3/4 as well as downregulated other BRD4 downstream targets such as MYC, but had relatively many off-targets. While not detected in the proteomics, GSPT1 and IKZF1 were also degraded with DC_50_s of ∼500 nM (5 h) and 200 nM (24 h) respectively.

##### 2.1.2.13 VHL

In a similar vein, Shah et al.^152^ attempted to design a covalent VHL binder. They aimed to replace the canonical hydroxyproline motif in VHL binders (making a hydrogen bond to Ser110 in VHL), with a sulfonyl fluoride group that could irreversibly bind the same serine. In two iterations they arrived at VHL-SF2 (Figure 17B) which showed an apparent IC_50_ = 35 μM in an FP competition assay, intact protein LC/MS showed a modest 65% labeling (100 μM; 24 h) of VHL, and a biotinylated version showed 44% binding in cells overexpressing VCB.

Based on this binder, the authors made BRD-SF2 (Figure 17B) a PROTAC targeting BRD4. In a HiBit assay, the PROTAC induced BRD4 degradation with DC_50_ = 17.2 μM; *D*_max_ = 60% (18 h). The PROTAC showed similar degradation of endogenous levels of the long isoform of BRD4. To prove a covalent mechanism in the cell, the author showed that degradation by BRD-SF2, though weak, is much more resistant to washout and competition by non-PROTAC VHL binders, compared to reversible PROTAC MZ1.^153^ To show generality, the authors made two AR targeting PROTACs. Both PROTACs showed degradation in a HiBit assay with DC_50_ in the 200–500 nM range but *D*_max_ ∼ 55%.

##### 2.1.2.14 FBXO22

A series of recent publications, reported almost simultaneously the discovery of FBXO22 as the effector ligase mediating degradation by electrophilic compounds.

**Figure 18:**
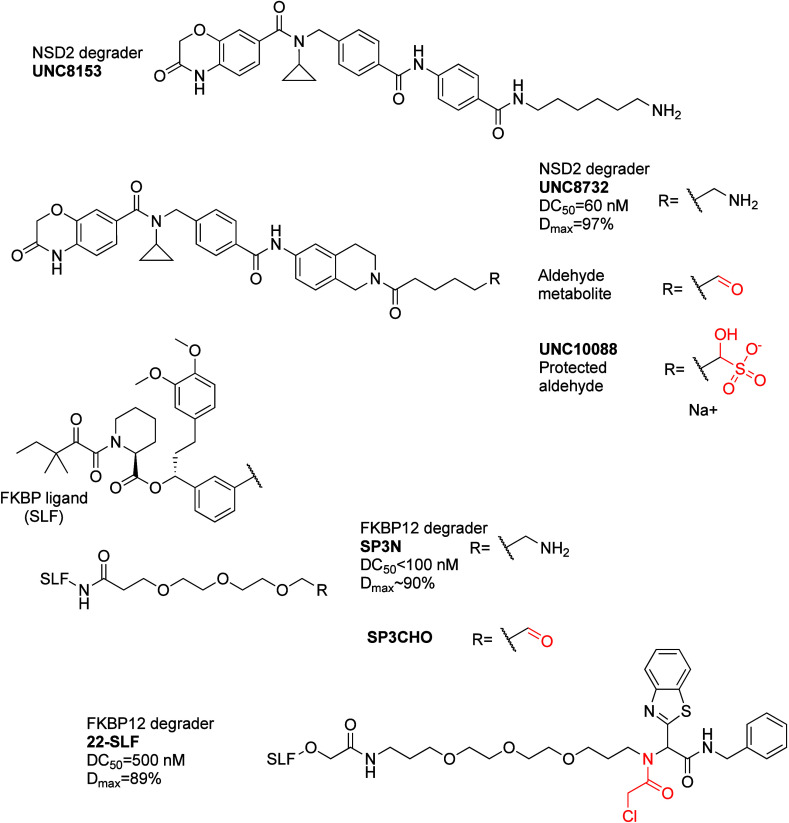
FBXO22 recruiting covalent PROTACs.

Hanley et al.^154^ had previously discovered UNC8153 (Figure 18), an alkylamine degrader of NSD2, a histone methyltransferase. In subsequent work^155^ they optimized the alkylamine linker to arrive at UNC8732 (Figure 18) with improved degradation properties (DC_50_ = 60 nM; *D*_max_ = 97%) which was extremely selective proteomically.

The authors hypothesized that the amine might be metabolized and is not the active species itself. They performed a metabolite identification study in cells treated with these amines and identified their aldehyde derivatives. The original compounds were depleted within 6 h.

The aldehyde was formed when UNC8732 was incubated in media containing 10% FBS even in absence of cells, but not in media lacking FBS. Aminoguanidine, a pan amino oxidase inhibitor, blocked the metabolism in the presence of FBS, suggesting that amine oxidases in the FBS metabolized the compound. In the absence of FBS in the media, no NSD2 degradation was observed, strongly suggesting the aldehyde is in fact the active species.

The authors made UNC10088 (Figure 18) a protected aldehyde bisulfite prodrug,^156^ this hydrolyses to the aldehyde in aqueous media fast and irrespective of FBS. In cells it degraded NSD2 irrespective of FBS and with comparable or better degradation parameters as the parent UNC8732, further underscoring the aldehyde version is the active degrader.

To identify the potential E3 ligase mediating the degradation they utilized the BioID approach.^157^ FBXO22 was among the few proteins enriched in the presence of UNC8732 (and a proteasome inhibitor). siRNA KD of FBXO22 rescued the degradation and NanoBret experiments validated UNC8732 induced proximity between FBXO22 and NSD2. The authors were also able to show compound mediated proximity and ubiquitination in an *in vitro* system (only for the aldehyde compound). A truncated molecule just containing the alkylamine moiety can compete for FBXO22 and reduce NSD2 degradation in response to UNC8732. They also showed that UNC10088 can dose-dependently stabilize recombinant FBXO22.

Using hydrogen–deuterium exchange mass spectrometry (HDX-MS) the authors localized compound binding to the region around cysteine 326. Indeed, a C326A mutant abolished the stabilization by UNC10088 (but did not affect protein stability in and of itself). The mutant also blocked compound induced *in vitro* proximity and ubiquitination. Using a washout experiment, where the protein thermal stability was measured in the presence or after washing out of the compound they showed the covalent interaction with the cysteine is reversible.

Finally, they showed that the recently reported XIAP alkylamine degrader^158^ likely operates through the same mechanism. XIAP degradation was sensitive to the presence of FBS as well as to amine oxidase inhibitors. Furthermore, addition of the NSD2 degraders could protect degradation of XIAP (presumably by competing for FBXO22). Finally, KD of FBXO22 also rescued XIAP degradation.

Kagiou et al.^159^ noticed that a series of SLF-alkylamine intermediates, exemplified by SP3N (Figure 18), that were prepared toward making a FKBP12 PROTAC library, were able in and of themselves to degrade FKBP12. The degradation could be rescued by competition with SLF, or by acylation of the free amine. Global proteomics showed exquisite selectivity of SP3N for FKBP12 degradation.

A CRISPR/Cas9 screen in an FKBP12 reporter cell line identified KO of the substrate receptor FBXO22 in cells that were resistant to SP3N degradation. Validation experiments showed that FBXO22 KO in another cell line also rescued degradation, moreover, re-expression of FBXO22 in the KO cell line restored degradation by SP3N. Co-IP experiments of HA-tagged FBXO22 and Flag-tagged FKBP12, showed a dose-dependent interaction between the two proteins, exclusively with SP3N treatment.

Similar to Nie et al.^155^ the authors showed that degradation only occurred in media with 10% FCS, suggesting a similar oxidation of SP3N to the active aldehyde species. In the presence of FCS the authors were able to detect the aldehyde SP3CHO (Figure 18) after 6 h by UPLC-MS/MS. Direct synthesis of SP3CHO showed improved degradation in cells independent of FCS. Using the NanoBit assay, SP3CHO induced ternary complex irrespective of FCS, whereas SP3N required FCS to do so. The same trend repeated in an *in vitro* ubiquitination assay with recombinant FBXO22.

Intact mass spectrometry of FBXO22 with SP3CHO identified the expected covalent adduct. Interestingly, conversion of the electrophile to a chloroacetamide (see below), or acrylamide, did not result in FKBP12 degradation.

Out of five cysteine mutants, only C326A rescued degradation. NanoBit confirmed no ternary complex is formed with the C326A mutant, nor was it able to ubiquitinate FKBP12 *in vitro*, or form an adduct with SP3CHO by MS. Proteome wide TMT-ABPP^15^ was able to identify 30% modification of FBXO22 C326 (in lysates spiked with recombinant SKP1-FBXO22) with relatively few significant off-targets.

The authors reproduced the FBXO22 dependent degradation of NSD2^154^ and XIAP^158^ alkyl amine degraders. Interestingly JQ1 alkylamine derivatives failed to degrade BRD4, suggesting optimization is still required for its recruitment.

Basu et al.^160^ developed a CRISPR/Cas9 activator screening platform, to enable identification of E3 ligases that can mediate degradation but their discovery is mitigated by low expression. They generated FKBP12 reporter cell lines with either high or low expression, and identified a PROTAC, 22-SLF (Figure 18), with no effect in the high-expression cell line, and a mild effect (∼30% degradation) in the low-expression cell line. They used the CRISPR activation screen to look for sgRNAs that enhance the activity of the PROTAC. The screen enriched two distinct sgRNAs, both activating FBXO22.

To validate FBXO22, they stably expressed FLAG-tagged FKBP12 and HA-tagged FBXO22 in HEK293T cells. 22-SLF was only able to degrade FLAG-FKBP12 in the presence of HA-FBXO22. The degradation was sensitive to preincubation with a proteasome inhibitor, neddylation inhibitor and SLF. 22-SLF showed a DC_50_ = 500 nM, *D*_max_ = 89% and rapid degradation of FKBP12 within 2 h. In three different cell-lines 22-SLF was able to degrade endogenous FKBP12 (after 24 h), which was abrogated on FBXO22 KO.

To assess the engagement of the electrophilic moiety, the authors synthesized 22-Biotin, and were able to enrich with it FBXO22 from cells expressing HA-tagged FBXO22, as well as endogenous FBXO22 that was competed by 22-SLF.

Using isoDTB ABPP,^13^ the authors identified four cysteines in FBXO22, one of which, C228 showed ∼20% occupancy for 22-SLF (among quite a few other cysteines interacting with the molecule). The low engagement was not due to low permeability but probably rather due to low affinity to FBXO22. Overexpressing a C228A FBXO22 mutant, rescued 22-SLF mediated degradation partially, the author noticed a second proximal cysteine 227 in the protein. C227A also partially mitigated degradation, and the double mutant C227A/C228A completely abolished it. Co-IP experiments showed that 22-SLF induced a ternary complex between WT FBXO22 and FKPB12, but not with the double cysteine mutant FBXO22. Three other mutations predicted by modeling to block compound binding had no effect on degradation, suggesting that the binding is mainly driven by reactivity of the electrophile.

To demonstrate generality the authors synthesized 22-JQ1 targeting BRD4 and 22-TEA, based on a specific ligand for the ALK kinase.^161^ Both PROTACs showed degradation of their targets at 2 μM that was abolished in FBXO22 KO cells. Finally, the authors show that switching the electrophile of 22-SLF into the less reactive acrylamide, can also facilitate FKBP12 degradation (even slightly better) which is surprising, but perhaps points to way to future optimization of a more selective recruiter.

### 2.2 Covalent DUBTACs

**Figure 19:**
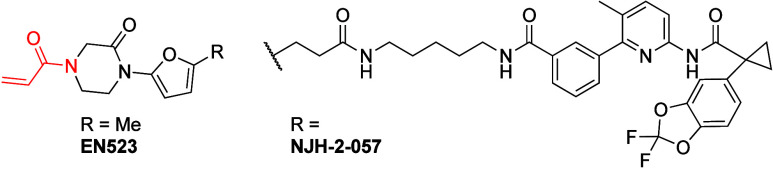
Structures of OTUB1 recruiter and lumacaftor based CFTR stabilizer.

The Nomura group introduced the concept of deubiquitinase targeting chimeras (DUBTACs) as a new modality for protein stabilization.^162^ Analysis of hundreds of chemoproteomics experiments led them to focus on the DUB OTUB1. They screened 702 covalent ligands against recombinant OTUB1. The top hit EN523 (Figure 19) was shown by LC-MS/MS to bind C23 (and not the catalytic C91). *In vitro* deubiquitination assays showed EN523 did not affect DUB activity. SAR showed the acrylamide was crucial for binding, and identified the furan ring as an exit vector for a linker. An alkyne derivative retained *in vitro* binding and was able to pull-down endogenous OTUB1 from cells.

As a target, the authors chose ΔF508-CFTR. This frameshift mutation in CFTR, is the most common mutation leading to the cystic fibrosis phenotype, causing protein destabilization, and proteasomal degradation before trafficking of the channel from the ER to the cell surface. Lumacaftor, is a small molecule chaperone that stabilizes ΔF508-CFTR and increases its trafficking to the membrane. The authors linked EN523 and lumacaftor resulting in NJH-2-057 (Figure 19) which maintained binding to OTUB1 *in vitro*. They tested its effect in ΔF508-CFTR expressing human bronchial epithelial cells. While lumacaftor, or EN523 had no effect, NJH-2-057 showed significant increase in CFTR protein levels. Another SAR library exploring various linkers was able to find both linkers that abolished the effect on CFTR as well as linkers that greatly improved it. The reduced version of NJH-2-057, was incapable of stabilizing CFTR, underscoring the importance of the covalent binding to OTUB1.

Global proteomic analysis validated that CFTR was stabilized by almost 8-fold compared to DMSO treatment, in a relatively selective fashion. An isoTOP-ABPP experiment did not identify many off-targets for NJH-2-057, but also showed a very low competition ratio for OTUB1 C23 (1.6) which may indicate low occupancy of OTUB1 is sufficient to drive the phenotype.

*In vitro* native-MS experiments showed evidence of the complex of OTUB1 and the relevant ΔF508-CFTR domain, only in the presence of NJH-2-057 but not for DMSO or EN523. Further, CFTR stabilization in cells was reduced by preincubation with either EN523 or lumacaftor, as well as by KD of OTUB1. Finally, to show that CFTR stabilization translates to improved function, the authors measured transepithelial conductance in primary human cystic fibrosis donor cells, following treatment with DMSO, lumacaftor or the DUBTAC. The latter showed significantly improved conductance over both DMSO and lumacaftor.

To show generalization, the authors synthesized four DUBTACs targeting Wee1 kinase using various linkers. Two of the four showed significant stabilization (1 μM; 24 h) that was of a similar magnitude to that seen with bortezomib treatment, whereas the Wee1 binder alone, or EN523, had almost no effect.

EN523 was further used to demonstrate stabilization of transcription factors (TF) by so-called TF-DUBTACs.^163^ EN523 was linked by copper free click-chemistry^164^ to an azide functionalized DNA hairpin encoding TF binding sites. For example, FOXO–DUBTAC #6 was able to stabilize FOXO3A (but not FOXO1, presumably due to a different ternary complex or site of ubiquitination). This resulted in elevated expression of target genes such as p27 and BIM. These effects were not seen in OTUB1 KO cells. Similar (though to a lower extent) effect was seen with TF-DUBTACs targeting p53 and IRF3.

### 2.3 Covalent PHICs

Siriwardena et al.^165^ introduced the concept of phosphorylation inducing chimeras (PHICs) through bifunctional molecules that induce proximity between a kinase and a neo-substrate. To do so, they linked allosteric kinase binders on the one hand with target binding ligands on the other. The number of allosteric kinase binders is limited, whereas there are many more available kinase inhibitors, however in order to induce phosphorylation the kinase active site must not be inhibited.

**Figure 20:**
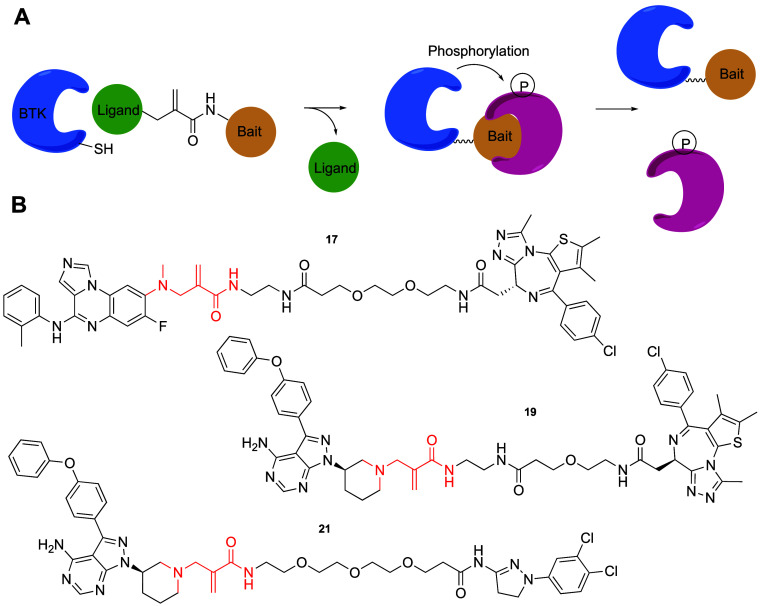
CoLDR based phosphorylation inducing chimeras (PHICs). (A) Schematic for the mechanism of action of covalent ligand directed release (CoLDR) based PHICs. (B) Chemical structures of **3** BTK targeting PHICs. While **17** and **19** recruited BTK to phosphorylate BRD4, **21** recruited ABL to phosphorylate BTK itself.

To overcome this obstacle. Pergu et al.^166^ employed the CoLDR chemistry we developed^79^ to install a target recruiting ligand onto BTK while releasing the BTK guiding inhibitor. They tested two CoLDR-PHICs based on BTK covalent inhibitors (**17**, **19**, Figure 20), using (S)-JQ1 as the target recruiter, or (R)-JQ1 as a negative control. In cells overexpressing BTK and BRD4 they could detect pTyr on HA-tagged BRD4 after immunoprecipitation, only for the (S)-JQ1 compounds. They were also able to show co-IP of BTK-FLAG with HA-BRD4 only upon treatment of the (S)-PHICs.

Next, they used a similar chemistry to install an ABL recruiter onto BTK, surprisingly, this PHIC, **21** (Figure 20), in cells transfected with both kinases, resulted in increased phosphorylation of BTK with no change in the phosphorylation levels of ABL.

## 3 Covalent Molecular Glues

Monovalent proximity inducers (or molecular glues) are another class of protein proximity inducers with their own set of advantages and challenges as compared to bifunctionals such as described above. Their small size makes them more desirable from a physicochemical properties’ perspective and much more amenable for translational applications. They may target traditionally “unligandable” proteins by stabilizing low-affinity endogenous, or new nonendogenous interactions. Such gluing events may result in surprising perturbations of function. On the other hand, molecular glues lack the intrinsic modularity of chimeric molecules and until recently were mostly discovered serendipitously. Recently, elegant phenotypic screening design accelerates rational molecular glue discovery. The introduction of electrophilic centers into glues can assist both the probability of binding to either or both interaction partners as well as help elucidate the mechanism of action of phenotypically discovered glues.

### 3.1 14–3–3 Peptide Interactions

14–3–3 proteins are a family of seven conserved isoforms that act as key modulators of the function of numerous other proteins.^167^ To do this, they form protein–protein interactions with phosphorylated peptides in their client proteins. Such binding events direct and modulate many cellular processes. Hundreds of 14–3–3 clients have been identified so far. Therefore, the ability to pharmacologically control such interactions is alluring.^168^

Fusicoccin A (FC-A) is a fungal metabolite that stabilizes the interactions between 14 and 3–3 protein and phosphorylated client proteins.^169^ Therefore, acting as a molecular glue. The ability to glue peptide clients to 14–3–3 proteins offers a route to tackle very challenging targets and inspired wide interest in the design and discovery of such molecular glues. Nucleophilic residues near the peptide binding site offer the opportunity for covalent molecular glues.

#### 3.1.1 Useful Definitions for Evaluating 14–3–3 Glues

- CC_50_: half-maximum ternary complex formation. When the protein and peptide are kept at a fixed concentration and the glue is titrated at increasing concentrations the CC_50_ is the concentration at which 50% of the effect is observed.
- The stabilization factor SF or SF_100_ (for 100 μM compound) is defined as the apparent *K*_D_ for the peptide/14–3–3 interaction in DMSO/*K*_D,app_ in the presence of compound.
- α, the cooperativity factor, defined as the stabilization factor at saturating concentration of compound (or maximal SF).

#### 3.1.2 Imine Based 14–3–3 Glues

Wolter et al.^170^ reported the first imine-based screen to identify reversible covalent molecular glues between 14 and 3–3 proteins and their client peptides. They identified Lys122 as a favorable target. It is located in a hydrophobic region of the peptide binding groove, as part of the binding pocket of FC-A. Moreover, computational analysis predicted it to have one of the lowest p*K*_a_ values (∼10) of the 18 lysine residues in 14–3–3σ. As a client peptide they chose one derived from the p65 subunit of NF-κB.

**Figure 21:**
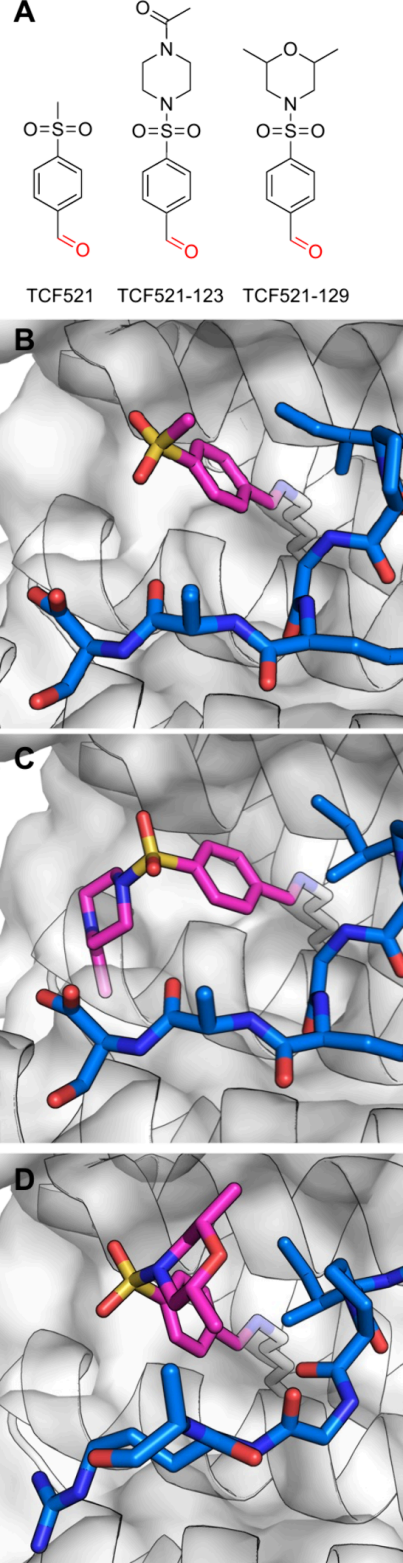
Imine based glues for the 14–3–3σ/p65 interaction. (A) Chemical structures of aldehyde glues. (B–D) Co-crystal structures of 14–3–3σ (white) with a p65 derived peptide (blue) and imine covalent glue (magenta). (B) TCF521; PDB 6YOW. (C) TCF521-123; PDB 6YPY. (D) TCF521-129; PDB 6YQ2.

A pilot crystallography soaking screen of 10 aldehyde fragments into crystals of the p65/14–3–3σ complex, observed density for three fragments bound to Lys122. Replacing the aldehyde of TCF521 (Figure 21A,B) with an acid, alcohol, amine, ketone or methyl resulted in loss of density in the crystal, suggesting imine formation was a key binding determinant.

They proceeded to screen 34 benzaldehyde analogues by crystallography. Only fragments with an electron withdrawing group, activating the aldehyde, resulted in electron density, but specifically bound to Lys122. Extending compound TCF521 into TCF521–123 or TCF521–129 (Figure 21A), resulted in improved binders that also showed imine formation by crystallography and by intact protein LC/MS (following reduction of the imine). Interestingly, although chemically similar the two sulfonamides adopted opposite conformations in the crystal (Figure 21C,D).

The compounds induced stabilization of the complex of 14–3–3 with p65 as assessed by FP. Compared to the DMSO control (*K*_D_ = 8.6 μM), at 1 mM compound TCF521–123 showed 3-fold stabilization and TCF521–129 showed 8-fold stabilization. Combined with the structures suggesting that TCF521–129 makes more extensive interactions with the p65 peptide, whereas TCF521–123 engages more with 14–3–3 the authors hypothesize, that increased contact with the client contributes more to the stabilization and provides the best starting points for molecular glues.

In terms of selectivity, these aldehydes were not able to stabilize interactions with TAZ, ERα, or p53 peptides. The latter two, the authors suggest, are incompatible due to polar interactions between the client and Lys122.

Cossar et al.^171^ followed up with a similar approach now targeting the 14–3–3/Pin1 interaction. They identified the relevant Pin1 phosphopeptide, and showed by crystallography it adopts a binding mode that allows small molecule binding to Lys122. They screened a library of 42 aldehyde fragments against this interaction via FA.

**Figure 22:**
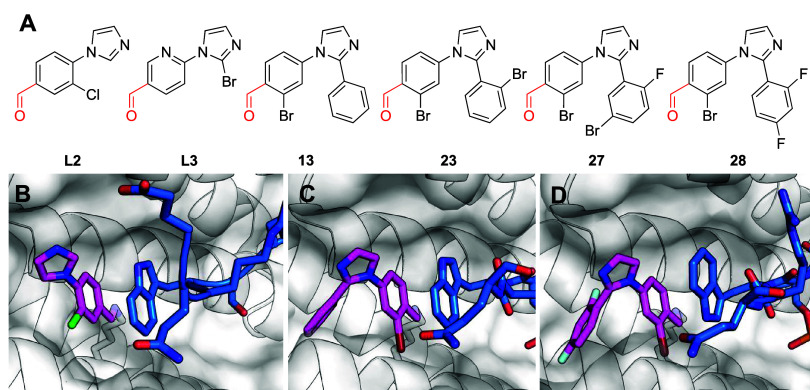
Imine based glues for 14–3–3σ/Pin1 interaction. (A) Chemical structures of aldehyde glues. (B–D) Co-crystal structures of 14–3–3σ (white) with a Pin1 derived peptide (blue) and imine covalent glue (magenta). (B) L2; PDB 7AXN. (C) 13; PDB 7BDT. (D) **28**; PDB 7BFW.

Eleven compounds were found to stabilize the complex. Co-crystallography elucidated the binding mode of two similar fragments L2, L3 (Figure 22A,B). The fragment binding to the lysine induced a rotamer flip to a nearby Trp residue that now formed stacking interactions with the fragments.

Two rounds of optimization led to compound **13** (Figure 22A,C) with CC_50_ = 101; *K*_D,app_ = 2.16 μM and SF_100_ = 10.8. Interestingly, it elicited improved stabilization of the ternary complex formation relative to the analogous 2-OH derivative (*K*_D,app_ = 3.92 μM, SF_100_ = 4.9), despite the latter having a 5-fold lower CC_50_.

A final library of phenyl substitutions yielded the best stabilizers with compounds **23**, **27**, and **28** (Figure 22A,D), eliciting CC_50_ values of 24, 118, and 79 μM, respectively. These showed a significant shift in apparent *K*_D_ ranging from low to submicromolar activity (1.15–0.27 μM) and SF_100_s ranging from 13- to 97-fold stabilization.

In DSF as an orthogonal assay, five equivalents of the Pin1 peptide did not elicit thermal stabilization, nor did compounds **27** or **28** by themselves. However, in the presence of both peptide and compounds a 1 and 2 °C shift was observed, respectively. In terms of cooperativity (α, or maximal stabilization) while **13**, **23**, and **27** showed α ∼ 60, **28** showed α = 270. Interestingly, FC-A reaches saturation of stabilization already at the lowest concentration tested of 8 μM, but only elicits a modest stabilization of 10-fold.

To assess selectivity, the authors tested stabilization of 13 different 14–3–3 client peptides, differing in the +1 amino acid (Trp in Pin1). The fragments showed no stabilization of peptides with polar +1 residues, but 28 did elicit 10–15 fold stabilization toward Raptor (Tyr), CFTR (Val) and Abl1 (Leu). The fragments did not compete for binding with these peptides, suggesting very low intrinsic affinity for the 14–3–3 on its own.

In follow-up work Verhoef et al.^172^ characterized the kinetics of stabilization of the Pin1/14–3–3 interaction by fragment **28**. They showed by time-dependent FA, that the binary complex is formed in under 10 min and is stable over time. The same goes for FC-A which shows rapid 10-fold stabilization that is stable over time. In contrast, **28** shows some stabilization at the first time point (∼6 fold), but then takes 2–3 h to reach full stabilization (∼20-fold).

The authors attributed this time dependency to the formation of the imine bond. To test that, they repeated the assay under different pH. The binary and FC-A complexes were indifferent to pH, whereas **28** displayed increased stabilization with higher pH (up to 48-fold stabilization at pH 8), consistent with imine bond formation behavior. Similar results were obtained an aldehyde fragment stabilizing the 14–3–3/p65 interaction.

Kinetic native mass spec measurements suggested that the Pin1 peptide first binds to the 14–3–3 followed by binding of the aldehyde. Combined with the time-dependent FP data, the authors suggest a model where the aldehyde first binds noncovalently and provides a certain amount of stabilization, which is then increased as the imine bond is formed over time.

#### 3.1.3 Disulfide Based 14–3–3 Glues

Disulfide tethering has a long history in site-specific fragment-based discovery.^173−175^ Sijbesma et al.^176^ used it to identify disulfide molecular glues for the 14–3–3/ERα interaction. They targeted the FC-A binding site at the interface of 14–3–3σ and the client phosphopeptide derived from ERα. 14–3–3σ is the only isoform that contains a native cysteine (C38) near the peptide binding site. In addition to this, they made two additional screening constructs in which they mutated out C38 and introduced a non-native cysteine at positions 42 or 45. All three constructs were screened by LC/MS in the absence of peptide or in a complex with a 15-mer ERα phosphopeptide, against a 1600-member disulfide fragment library under mild reducing conditions (100 μM βME).

Hits were annotated as competitive if they only hit in the absence of peptide, as cooperative if they showed preferential labeling to the protein-peptide complex, or neutral if they hit both. The three sites produced different ranges of potency (% labeling) and cooperativity with C38 showing the lowest potencies (maximal labeling <55%) but high fraction of cooperative hits, and C45 showing many hits with >75% labeling against both bound and *apo*. C42, yielded intermediate results.

**Figure 23:**
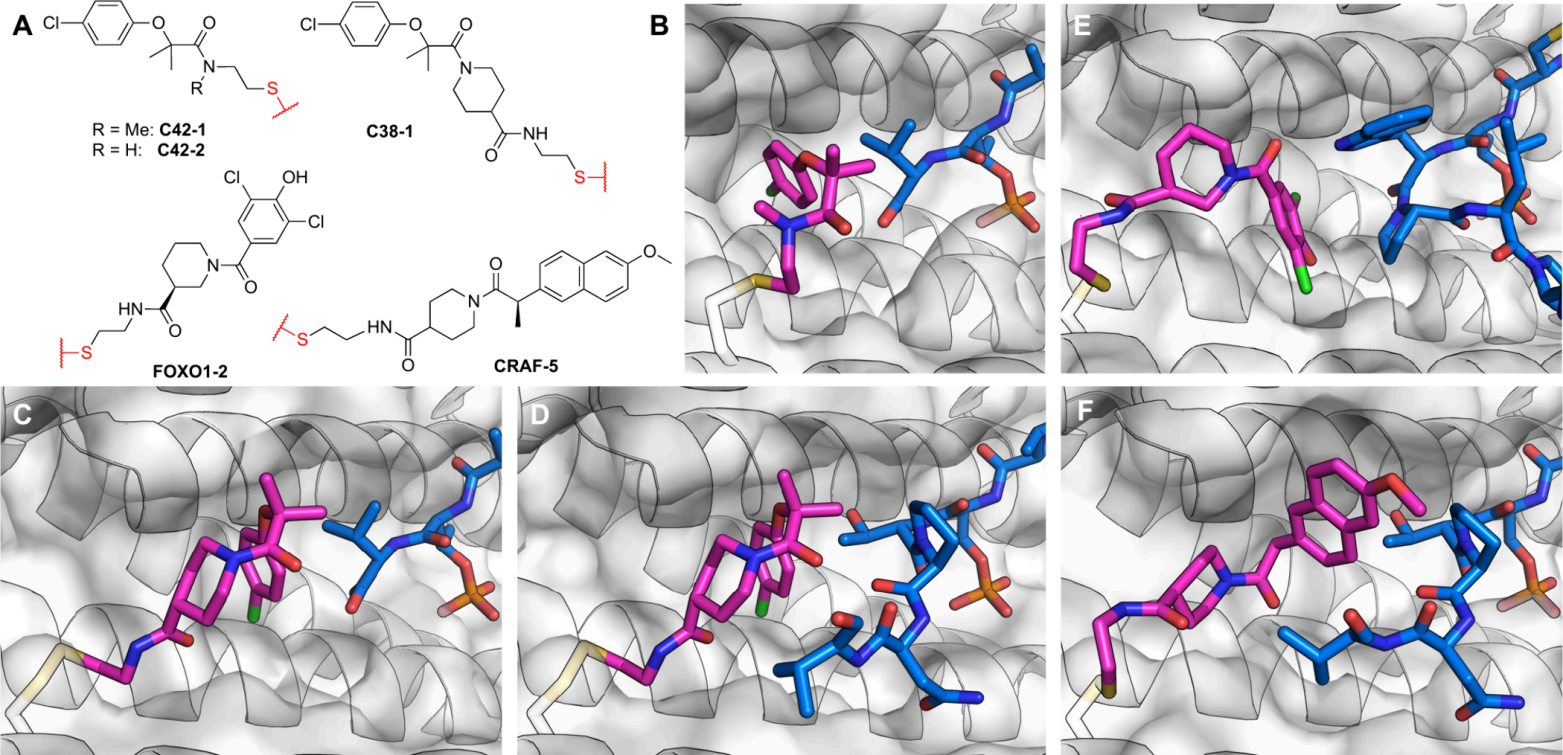
Disulfide 14–3–3 glues. (A) Chemical structures of disulfide stabilizers of 14–3–3 client peptides. (B–E) Co-crystal structures of 14–3–3σ (white) in complex with client peptides (blue) and disulfide glues (magenta). (B) ERα; C42–1; PDB 6HHP. (C) ERα; C38–1; PDB 8AFN. (D) C-RAF; C38-1; PDB 8AV0. (E) FOXO1; FOXO1-2 PDB 8A62. (F) C-RAF; CRAF5; PDB 8A68.

For C42, the most cooperative fragment was C42–1 (Figure 23A,B); demonstrating a 2.3-fold increase from 26% (*apo*) to 60% (bound). Several hits were confirmed in LC/MS dose–response experiments. Both C42–1 and C42–2 (Figure 23A) showed strong binding preference to the complex over the 14–3–3σ alone. Labeling by C42–2 was improved ∼300-fold, to EC_50_ = 3 μM for the complex. Fragment C42–1, retained >80% labeling to the complex even at 100 nM fragment and 1 mM βME. Cooperative binding was less pronounced for the other primary screening hits.

By FP, in the absence of fragment *K*_D,app_ was 1.3 μM. This decreased to 32 nM in the presence of C42–1 (100 μM), 92 nM in the presence of C42–2, and 4.2 nM for the positive control FC-A, representing 40- and 14-fold stabilization by the disulfide fragments.

Crystal structures of C42–1 and C42–2 soaked into the complex, suggested that noncovalent interactions drove the binding event (Figure 23B). This was further supported by NMR observed binding experiment of the noncovalent fragment.

In terms of client selectivity, peptides from ExoS, TAZ and TASK3 were evaluated. Fragment C42–2 labeling of 14–3–3σ(C42) was enhanced in the presence of TASK3 to a similar degree as by ERα (7 μM and 3 μM respectively). Peptides derived from ExoS or TAZ however, predicted to clash with fragment binding based on the peptide binding mode, indeed inhibited binding of C42–2, compared to the *apo* protein labeling, FP showed similar results, altogether suggesting the potential for client selectivity (albeit in this non-natural introduced cysteine system).

In subsequent work Sijbesma et al.^177^ further characterized three of the hits against the engineered C45. One of which showed increased binding to 14–3–3σ(C45) in the presence of the ERα peptide (from ∼50% labeling of *apo* to ∼90% labeling of the peptide-bound). The other fragments bound the protein both in the presence or absence of peptide. While crystal structures validated the fragments binding in the vicinity of the peptide, neither fragment showed peptide binding stabilization, especially peculiar for the fragment that showed increased binding in the presence of the peptide.

Kenanova et al.^178^ followed up with more systematic disulfide screens for five additional targets, now only going after the native C38 in 14–3–3σ. The targets represent different interaction modes with 14–3–3 “truncated” (ERα), “turned” (FOXO1), or “linear” (C-RAF, USP8, SOS1).

The peptides were screened at 2× their reported *K*_D_ values with fixed (100 nM) concentration of 14–3–3σ. The complexes were incubated with 200 μM fragments. Hits were defined as showing % labeling higher than three standard deviations over the average library labeling rate. Between 8 and 23 hits were found for all five proteins.

Hit compounds were further profiled via: (1) DR_50_ - Dose response of labeling via intact protein LC/MS (fixed protein and peptide concentration), (2) EC_50_, Dose response of peptide binding via FP (fixed protein and peptide concentration), and (3) *K*_d,app_, with fixed concentration of compound (1 mM) and peptide, and varying concentrations of 14–3–3.

Compound C38–1 (Figure 23A) was identified as top hit for ERα, C-RAF, and USP8. In all three assays it showed a marked preference for C-RAF, followed by ERα and USP8, and had no activity with FOXO1 or SOS1. C38–1 showed DR_50_ values of 7 nM, 24 nM and 18.1 μM for C-RAF, USP8 and ERα respectively. EC_50_ values were similar for the three proteins (ranging from 0.9 to 3.4 μM). In terms of stabilization (at 1 mM) C38–1 stabilized peptide binding by 81-fold, 19-fold and 4-fold for C-RAF, ERα, and USP8 respectively. Co-crystal structures of C38–1 in complex with all three peptides were determined and showed that for ERα it occupied a similar binding mode as the previously discovered stabilizer for the N42C mutation, where the longer linker to the disulfide now allowed reaching to C38 (Figure 23C,D).

Eight of the FOXO1 screening hits were validated by MS dose response. Compound FOXO1–2 (Figure 23A) showed a DR_50_ = 360 nM, with >2 mM DR_50_ to the apo protein. However, compared to these dramatic selectivity to the peptide bound complex, it showed only mild 5-fold stabilization, possibly due to the already high-affinity of the phosphopeptide (*K*_D_ = 50 nM) which nears the assay detection limit. Co-crystal structures of FOXO1–2 with the complex, showed a Trp residue at position +1 flipped to form a hydrogen-bond with the amide carbonyl (Figure 23E). Smaller residues could not reach the fragment perhaps explaining its marked selectivity.

From the C-RAF screen, 11 compounds showed a similar scaffold, which was also analogous to the FOXO1 stabilizer scaffold although with a different linker. Nine of the 16 selective compounds were validated by MS, four of which showed activity by FA, with DR_50_ in the range of 0.6–12.2 μM, EC_50_s of 0.2–13.5 μM and stabilization of 77–426-fold (at 1 mM compound). Compound CRAF-5 (Figure 23A) for example showed a 246-fold stabilization (*K*_D_ = 23 μm to 92 nM). Structures of these selective C-RAF stabilizers (Figure 23D,F) showed differences in conformation of the peptide/compound interactions, highlighting the flexibility of the peptide.

#### 3.1.4 Dual Covalent 14–3–3 Glues

**Figure 24:**
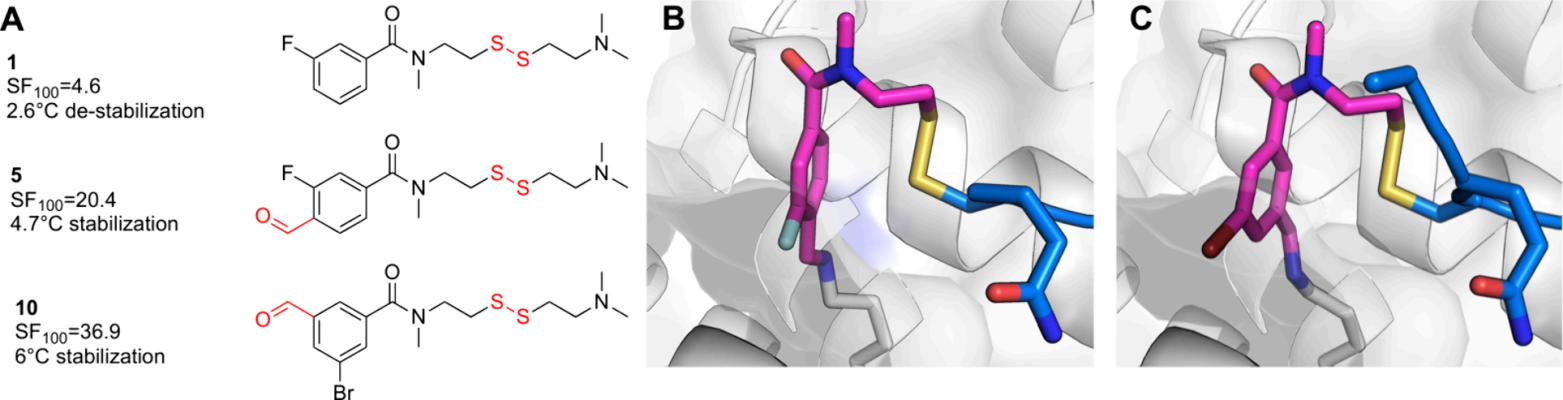
Dual covalent glues for 14–3–3 proteins. (A) Chemical structures of parent disulfide glue **1** and dual-covalent derivatives **5** and **10** that mediate an interaction between **14** and 3–3γ and ERRγ. (B,C) Co-crystal structures of the ternary complex between **14** and 3–3σ (white), ERRγ peptide (blue) and dual covalent glues (magenta) interacting with a cysteine on the client peptide and Lys122 on 14–3–3. (B) **5**; PDB 8B4Q. (C) **10**; PDB 8B5P.

Somsen et al.^179^ combined a fragment that forms a disulfide bond with C180 of the ERRγ phospho-peptide with a fragment that forms an imine with Lys122 of 14–3–3σ. They evaluated the stabilization of the ERRγ/14–3–3γ complex by the dual covalent glues (termed “molecular locks”) by FP in the absence or presence of 100 μM compound (24 h incubation). The fragment containing only the disulfide (1; Figure 24A) demonstrated a 4.6-fold stabilization, whereas the fragment containing the aldehyde alone showed none. Dual-covalents showed 7–37-fold stabilization.

They further characterized the complexes by differential scanning fluorimetry (DSF). The compounds that showed the highest stabilization in the FP assay, **5** and **10** (Figure 24A) also increased the 14–3–3γ melting temperature by 4.7 and 6.0 °C, respectively. The disulfide parent **1** (Figure 24A) decreased the melting temperature by 2.6 °C. Intact protein LC/MS (following reduction of the imine) and cocrystal structures confirmed both covalent bonds were formed (Figure 24B,C).

To explore the role of the disulfide bond formation in stabilization, the authors showed that high concentration TCEP (reducing the disulfide bond) abolished both the peptide binding stabilization as measured by FP as well as the thermal stabilization. Similar results were obtained for a ERRγ C180S mutant, abrogating disulfide bond formation. To assess the role of the imine bond formation, they analyzed the effect of pH on peptide *K*_D_. The peptide *K*_D_ in the absence of a molecule or in the presence of just the disulfide compound **1** were unaffected by pH. Compounds **5** and **10**, however, demonstrated worse apparent *K*_D_ as the pH decreased, until at pH 6.5, they converged to the stabilization of disulfide **1**. This suggests the imine formation is also an important factor in the stabilization.

In terms of selectivity, when tested against eight additional 14–3–3 binding peptides, compounds **5** and **10** proved highly selective for ERRγ with **5** showing minor stabilization of SOS1 and Pin1 derived peptides, and **10** also showing 8-fold stabilization of RND3 derived peptide. The latter contains a cysteine residue at the corresponding position to the ERRγ peptide. Indeed, the authors were able to determine a cocrystal structure showing **10** adopts an identical pose in complex with 14–3–3 and RND3 derived peptide, but only forms the disulfide bond and the major species in the crystal contains the unbonded aldehyde. It is also interesting to note that a peptide derived from PKR also contains a cysteine at the same position but did not elicit stabilization by either compound. The structure of 14–3–3σ with the PKR peptide, shows the terminal carboxylate salt bridges with Lys122 which may preclude imine formation.

To simulate the competitive cellular environment the author evaluated the effect of coincubation of 14–3–3 with eight client peptides in addition to ERRγ derived peptide, and measured tricomplex formation by LC/MS following reduction of the imine. After 7 h the mixture only inhibited the complex formation by 2-fold (from 70% to 35%). In addition, they evaluated the effect of HEK293 cell lysate on the preformed 14–3–3γ/ERRγ in absence or presence of compound **5**. The latter could withstand up to 2 mg/mL^–1^ cell lysate.

#### 3.1.5 Irreversible 14–3–3 Glues

Konstantinidou et al.^180^ aimed to improve the potency and selectivity of their previously discovered nonselective disulfide hit C38–1 (Figure 23A). To do so, they started with replacement of the disulfide tether with different electrophiles, connected through various linkers. While acrylamides and epoxides did not work, Chloroacetamides were able to both bind and stabilize client peptides.

**Figure 25:**
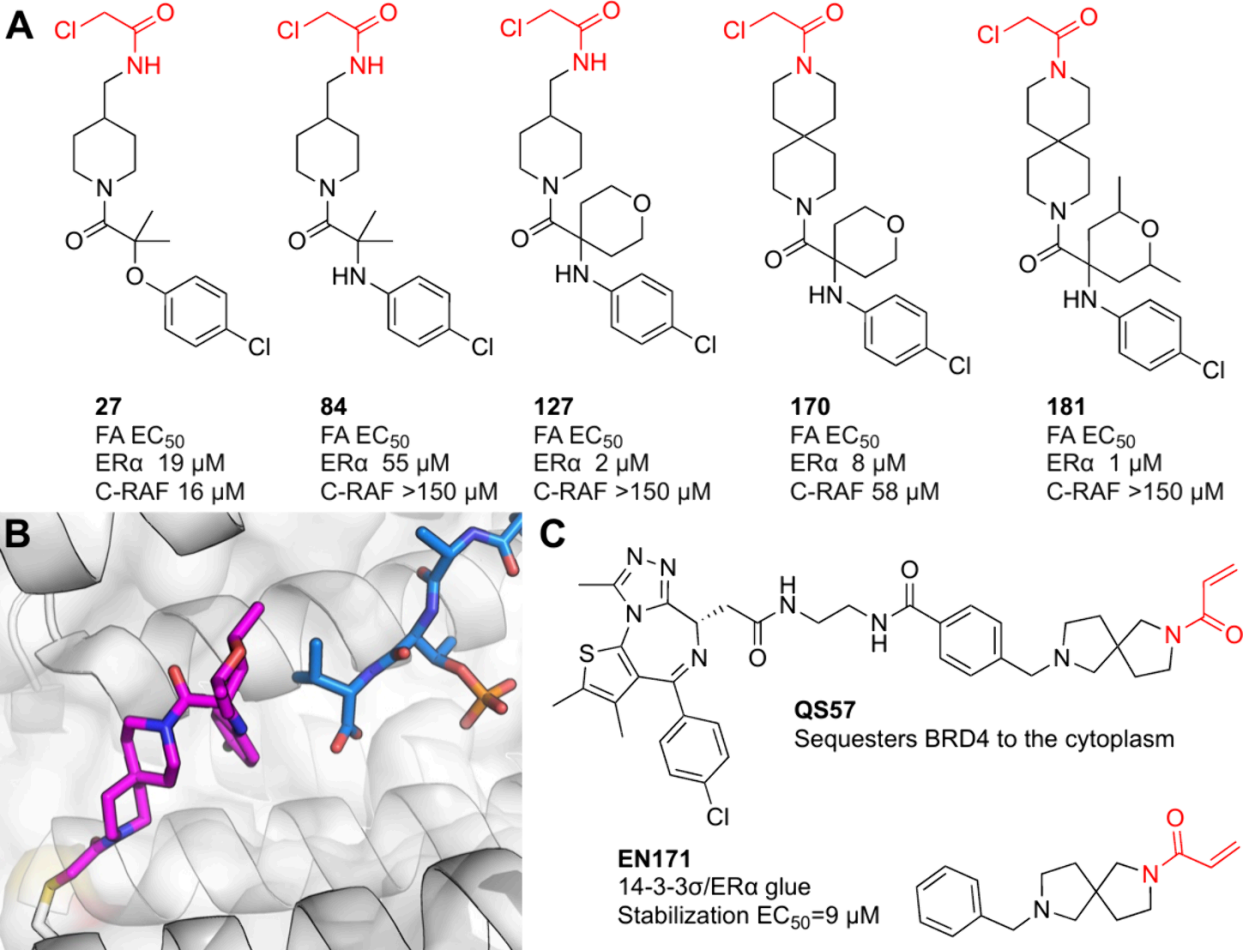
Irreversible 14–3–3/peptide molecular glues. (A) Medicinal chemistry optimization campaign of chloroacetamide glues for 14–3–3σ to ERα client peptide. (B) Co-crystal structure of 14–3–3σ (white), ERα client peptide (blue), and compound **181** (magenta). (C) Structures of an irreversible fragment that binds to 14–3–3σ and stabilizes its interaction with a peptide from ERα, and a bifunctional that recruits BRD4 to 14–3–3σ and therefore sequesters it in the cytoplasm.

A thorough structure-based medicinal chemistry campaign (Figure 25A) ultimately led them to compound **181**. This compound showed a marked SF_100_ = 116 stabilization of the ERα phosphopeptide (From 2 μM to 18 nM), with EC_50_ = 1 μM for stabilization. It could fully label 14–3–3σ within 1 h at low concentration, and showed robust selectivity against seven alternative client peptides including C-RAF which the parent compound C38–1 preferentially stabilized. 181 was comparable or better than FC-A in almost all stabilization parameters.

Shao et al.^181^ reported another attempt at irreversible stabilizers. They screened 875 irreversible fragments (125 μM) against the native Cys 38 in 14–3–3σ in an FP assay assessing the stabilization of binding of a phosphorylated ERα peptide substrate. They identified one acrylamide hit, EN171 (Figure 25B), that stabilized the interaction to a similar degree as FC-A. At 1 h incubation, EN171 showed an EC_50_ = 9 μM for stabilization of the ERα peptide. Out of 10 alternative client peptides, EN171 stabilized the binding of YAP, TAZ, CDC25B, and MYC with EC_50_ of 45–253 μM.

LC/MS/MS analysis revealed that EN171 binds C96 in addition to the predicted C38. Mutational studies showed that elimination of either cysteine alone did not prevent compound binding to the protein (at 50 μM) and both single mutants showed reduction of peptide binding stabilization.

To evaluate cellular engagement with 14–3–3σ the authors performed ABPP experiments that showed low competition ratios for both cysteines corresponding to 12–17% engagement, while 46 other cysteines showed more significant engagement. Competition proteomics experiments with an alkynylated version of EN171 in a lysate spiked with 14–3–3σ showed similar results, engagement with 14–3–3σ but modest selectivity.

Luciferase reporter assays for transcription of the ERα and Hippo pathways, indicated EN171 inhibited these activities with EC_50_s slightly above 10 μM. Knockdown of 14–3–3σ rescued this effect, suggesting on-target activity.

The authors then utilized the fact that 14–3–3 proteins are restricted to the cytoplasm. By making a bifunctional molecule QS57 (Figure 25B), that would bind 14–3–3 on the one hand (through EN171) and BRD4 on the other (through JQ1) they sought to sequester BRD4 from the nucleus to the cytoplasm. This is a novel concept for covalent bifunctionals, with some precedents with noncovalent bifunctionals.^182−184^ They were able to show such translocation to the cytoplasm at very high concentration (100 μM) of their bifunctional molecule, which was attenuated by knockdown of 14–3–3.

It is evident that the majority of covalent 14–3–3 targeting glues relies on reversible covalent chemistry (aldehydes, disulfides; Table 3) and these have yet to translated to cellular activity. Whereas one report on irreversible glues already shows modest cellular activity. The utility gluing 14–3–3 client peptides for chemical biology and translational applications will depend on the ability to progress these to cellular activity.

**Table 3:** Covalent Glues of 14–3–3/Peptide Interactions^a^

warhead	target residue	client	discovery	stabilization	selectivity^a^	cell activity	ref
aldehyde	K122	p65	crystallography soaking screen of 10 aldehydes	8-fold at 1 mM	3/3	NR	(170)
aldehyde	K122	Pin1	FP screen of 42 aldehyde fragments	97-fold at 100 μM	10/13	NR	(171)
				270 at 1 mM			
disulfide	N42C	ERα	disulfide tethering screen (1600)	40-fold at 100 μM	2/3	NR	(176)
disulfide	C38	CRAF	disulfide tethering screen (1600)	81-fold	2/5	NR	(178)
		ERα		19-fold			
		USP8		4-fold (1 mM)			
disulfide	C38	FOXO1	disulfide tethering screen (1600)	5-fold at 1 mM	0/4	NR	(178)
disulfide	C38	CRAF	disulfide tethering screen (1600)	246-fold at 1 mM	0/4	NR	(178)
dual	K122/C180 (client)	ERRγ	fragment merging	37-fold at 100 μM	5/8	complex could form in lysate	(179)
chloroacetamide	C38	ERα	structure based optimization	116-fold at 100 μM	5/7	NR	(180)
acrylamide	C38	ERα	FP screen of 875 electrophiles	6-fold at 100 μM	6/10	10–100 μM activity	(181)

a Number of alternative clients not stabilized out of the number tested.

### 3.2 CypA-K-Ras^G12C^

**Figure 26:**
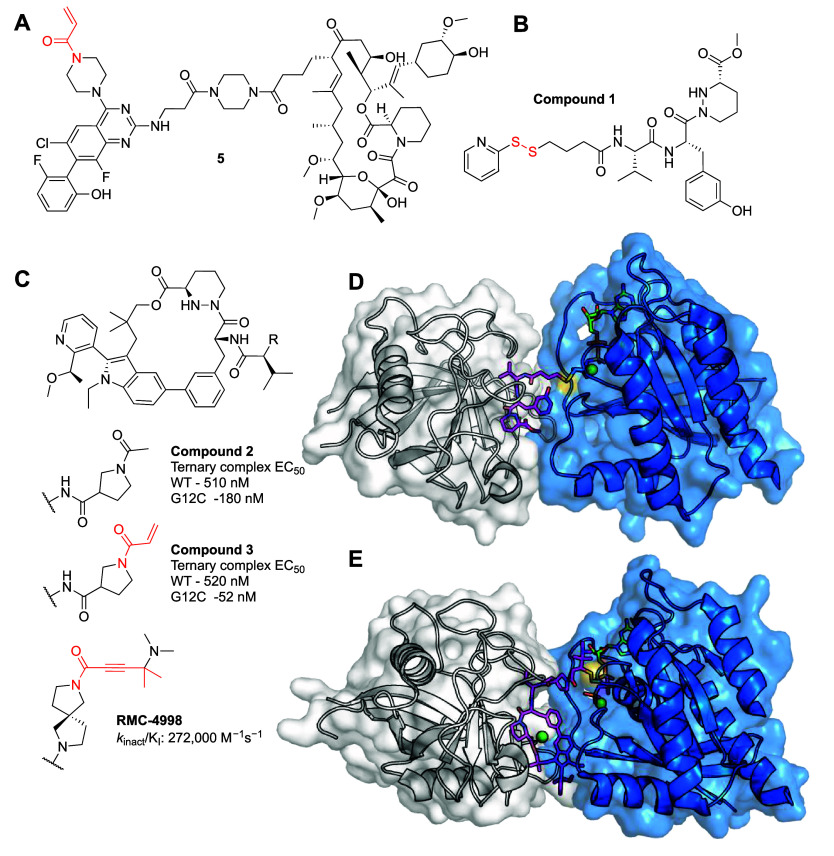
Covalent glues targeting K-Ras^G12C^. (A) Chemical structure of compound **5**. Despite being bifunctional, it labels K-Ras^G12C^ 4× better in the presence of FKBP12. (B,C) Chemical structures of covalent glues that mediated the interaction between CypA and K-Ras^G12C^. (D,E) Co-crystal structures of the ternary complex between CypA (white), K-Ras^G12C^ (blue) in its GTP (nonhydrolizable analogue in green) bound form and a covalent glue (magenta). (D) Compound **1**; PDB 8G9Q. (E) RMC-4998; PDB 8G9P.

Zhang and Shokat^185^ reported covalent bifunctional molecules that are able to bring into proximity K-Ras mutants with cyclophilin A (CypA) and FKBP12. They first made a series of compounds based on ARS-1620^23^ targeting K-Ras^G12C^ linked by various linkers to either SLF, FK506 or cyclosporin A (See for example compound **5** in Figure 26A). Not surprisingly these compounds maintained binding to FKBP12 and CypA (*K*_D_ < 200 nM). What was a little unexpected was that, while the compounds were able to covalently label K-Ras^G12C^ on their own, the labeling rate was enhanced in the presence of the cognate binding partner. For example, compound **5** labeled ∼4× more efficiently in the presence of 10 μM FKBP12, another compound labeled ∼2× more efficiently in the presence of CypA. This may suggest some intrinsic affinity between K-Ras and FKBP12/CypA. It is also interesting to note that unmodified ARS-1620 labeled better than any of the bifunctional derivatives regardless of the presence of a binding protein.

The authors note that compounds with long linkers (in this case 1,6-diaminohexyl) labeled K-Ras the slowest, perhaps due to the entropic cost^186^ this adds another incentive to aim for shorter linkers for bifunctionals in addition to better permeability and opportunities for cooperative binding.^187^

To validate the formation of ternary complex formation, the authors used size exclusion chromatography *in vitro*. In cells they were able to show partial engagement by pull downs of FKBP12/CypA followed by WB for K-Ras, that was, however, insufficient to block downstream MAPK signaling.

ARS-1620 preferentially binds to the inactive GDP-bound K-Ras^G12C^. To assess the effects of ternary complex formation on the GTP-bound K-Ras, two additional bifunctional ligands were designed, targeting the non-naturally occurring K-Ras mutation M72C.^188^ Similar to the G12C targeting compounds, these ligands also showed accelerated labeling of K-Ras^M72C^ in the presence of their cognate protein binders. That said, the overall labeling rates were still relatively slow and did not reach 50% labeling after 48 h. Still, in a TR-FRET assay to measure Ras/Raf complex formation, the ternary complex blocked association with full-length B-Raf (but not with just the Raf1 Ras binding domain).

More recently, a team from Revolution Medicines reported a covalent glue between CypA and K-Ras^G12C^.^189^ They started out with a minimal CypA-binding moiety derived from the natural product sanglifehrin A.^190^ and a promiscuous disulfide warhead (Figure 26B; compound **1**). Counterintuitively, this compound, with no specific K-Ras recognition incorporated into it, was sufficient to glue the two proteins, as demonstrated by a cocrystal structure (Figure 26D). Based on this structure, they ended up cyclizing the glue to fix its confirmation and build out additional molecular interactions with K-Ras.

The noncovalent compound 2 (Figure 26C) formed ternary complexes between CypA and K-Ras (GMPPNP bound) with an EC_50_ of 510 nM for the WT and 180 nM for the G12C mutant.

The covalent (acrylamide based) version of the same compound retained the same EC_50_ for K-Ras^WT^, but improved toward G12C to EC_50_ = 52 nM. Transition to an ynamide warhead improved its potency further EC_50_ = 28 nM, and its selectivity (39-fold more selective over WT). Optimizing the linker conformation (RMC-4998; Figure 26C) further improved the maximal rate resulting in *k*_inact_/*K*_I_ = 272,000 M^–1^ s^–1^. This represents a significant improvement over Sotorasib and Adagrasib.^26,27^ It is interesting to note that RMC-4998 bound to CypA reversibly with a relatively low-affinity *K*_D_= 1.09 μM.

Neither RMC-4998 nor CypA were able to bind to K-Ras^G12C^ alone. The compounds were also quite selective, both to the nucleotide bound state, (no complex formed with GDP-loaded K-Ras^G12C^), as well as against wild-type K-Ras, N-Ras, or H-Ras.

With regards to blocking Ras effectors, the tricomplex mediated by RMC-4998 dissociated the Ras interaction domains of B-Raf and RALGDS in a biochemical assay with single digit nM potency. In a live-cell kinetic assay, treatment with RMC-4998 led to rapid (minutes) association of K-Ras^G12C^ with CypA on the one hand and dissociation of full-length C-RAF on the other hand. This translated to potent inhibition of ERK signaling in cells with IC_50_ in the 1–10 nM range. Full labeling of K-Ras^G12C^ in cells occurred within 5 min, much faster than Sotorasib and Adagrasib, probably reflecting the fact that RMC-4998 binds the GTP bound form and does not need to “wait” for hydrolysis of the GTP.

Further optimization of RMC-4998 led to RMC-6291 that showed improved efficacy in cellular models and broad activity *in vivo* against a panel of PDX models. It is currently evaluated in the clinic.^191^

### 3.3 DCAF16-BRD4

**Figure 27:**
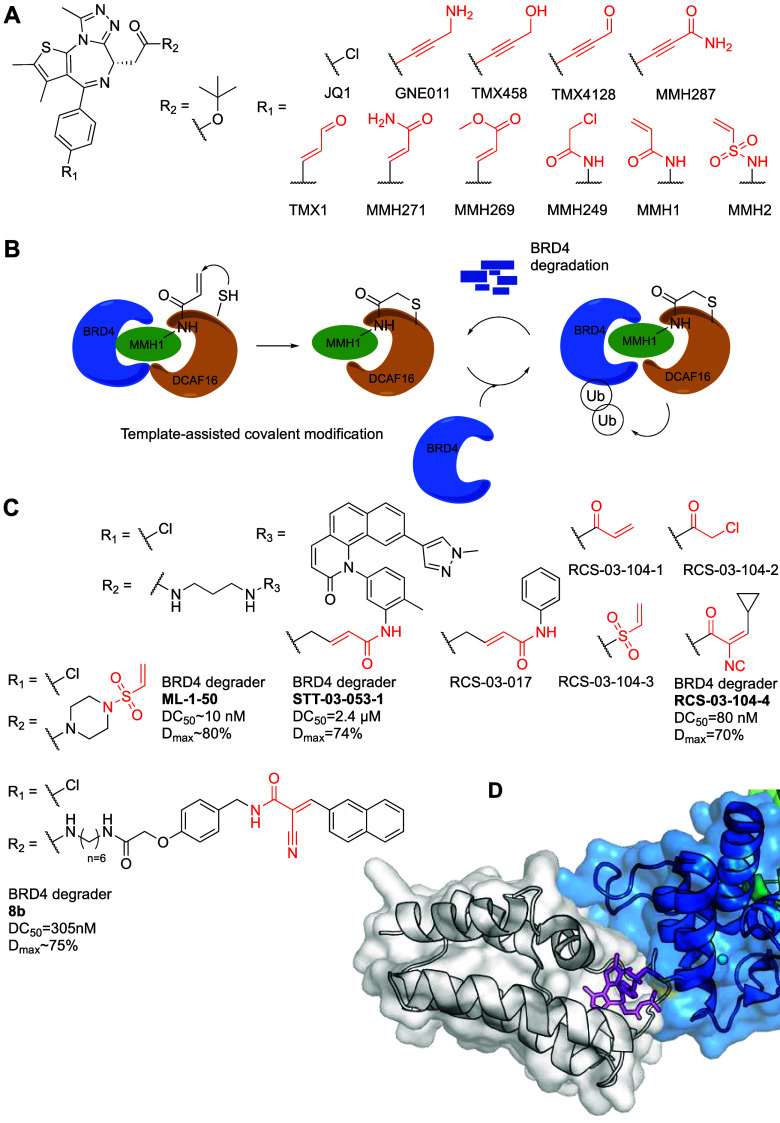
Covalent BRD4/DCAF16 glues and related DCAF16/11 PROTACs. (A) A series of monovalent BRD4 degraders recruiting DCAF16 via template assisted covalent modification. (B). Schematic representation of the degradation mechanism of action of BRD4 degradation through template assisted covalent modification of DCAF16. (C) Additional glues and bifuncational degraders of BRD4 that recruit DCAF16 through alternative cysteines or DCAF11. (D) Structure of the ternary complex between BRD4 (white), MMH2 (magenta) and DCAF16 (blue; PDB 8G46).

GNE011 (Figure 27A) is an alkynilated derivative of JQ1. It was reported to be able to monovalently degrade BRD4.^192^ Li et al.^110^ attempted to decipher its mechanism of action.

Using a reporter cell line, they showed that it induces selective degradation of the second bromodomain of BRD4 (BRD4_BD2_) with *D*_max_ = 50%. Through further analogueing, the authors identified TMX1 (Figure 27A), an acrylaldehyde derivative that improved *D*_max_ to 80%. The degradation was proteasome, and neddylation dependent.

To identify the underpining effector, the authors performed a CRISPR screen in the BRD4_BD2_ reporter cell line, using a library composed of components of the UPS. DCAF16 was unambiguously identified as required for degradation. It was corroborated through a KO cell line in which the compounds were no longer able to degrade BRD4 and through a viability resistance screen that also identified DCAF16 as the top hit. Moreover, IP-MS with BRD4_BD2_ as bait confirmed compound dependent direct interaction between BRD4_BD2_ and DCAF16. This direct interaction was corroborated by a TR-FRET experiment with fully recombinant proteins. The TR-FRET experiments also indicated that the interaction diminished at concentrations higher than 5 μM. Such a “hook effect” pattern is uncharacteristic for monovalent degraders, which suggested to the authors perhaps this is a result of a covalent interaction.

Upon incubation of DCAF16-DDB1 with TMX1, only 8% labeling was identified by intact protein MS, while adding BRD4_BD2_ increased the labeling to 50%. This result suggested that TMX1 binding to BRD4_BD2_ facilitated binding to DCAF16, in a fashion that positioned it to form a covalent bond with DCAF16. The authors named this: “template-assisted covalent modification” (Figure 27B). GNE11 showed a similar pattern albeit with lower labeling. The bifunctional KB02-JQ1 that was previously reported to bind DCAF16, bound regardless of the presence of BRD4_BD2_.

It should be noted that around the same time a second group also discovered DCAF16 as the ligase responsible for the degradation by GNE-011^193^ as well as a third group that showed DCAF16 mediates the degradation of BRD4 by another noncovalent bifunctional JQ1 derivative, that acts as a glue.^194^

The authors theorized that optimizing the electrophile might lead to improved complex formation and degradation. They discovered an acrylamide, MMH1, and a vinylsulfoneamide MMH2 (Figure 27A) that showed improved *D*_max_ = 95% and DC_50_ ∼ 1 nM. Moreover, in washout experiments these were superior to noncovalent degraders of BRD4. Reduced (nonreactive) versions of MMH1 and MMH2 showed negligible activity, further highlighting the necessity of covalent bond formation.

A cryo-EM structure of DDB1/DCAF16/MMH2/BRD4_BD2_ elucidated the template assisted covalent bond formation (Figure 27D). DCAF16 and BRD4_BD2_ display an interface of 560 Å^2^. MMH2 binds at the interface in the JQ1 binding site, and shows continuous density with C58 on DCAF16, suggestive of covalent bond formation. Alanine scanning of DCAF16 corroborated C58 as the covalent site required for the covalent glue degraders (but not KB02-JQ1) and intact protein LC/MS showed that the C58S mutant completely eliminated DCAF16-TMX1 covalent interaction.

In an accompanying manuscript Hassan et al.^195^ describe a wider series of covalent JQ1 analogues that was characterized for BRD4_BD2_ degradation, complex formation with DCAF16 and reactivity (Figure 27A). All compounds showed similar binding affinity to BRD4_BD2_ (as well as BRD_BD1_) as measured by competitive AlphaLisa.

TMX458, MMH269 and MMH271 were inactive, while all aniline based electrophiles were active with MMH1/2 significantly more so than chloroacetamide MMH249. Degradation was completely dependent on covalent bond formation, all noncovalent analogues lost this activity. TR-FRET measurements showed that only MMH1, MMH2 and TMX1 robustly formed a ternary complex, and to a lesser extent MMH249 and TMX4128. Nevertheless, compounds that did not show complex formation in this assay were still able to induce some degradation (such as GNE011 and MMH287).

As part of a separate campaign Sarott et al.^196^ made STT-03–053–1 (Figure 27C), a JQ1 based bifunctional targeting viral RNA replication that contains an acrylamide as part of the target binding moiety, connected to JQ1 through a different vector from the aforementioned glues. Surprisingly, it induced BRD4 degradation. In Jurkat BRD4-HiBiT cells it displayed DC_50_ = 2.4 μM and *D*_max_ = 74%. Reducing the acrylamide abolished the degradation. A CRISPR screen in K652 cells again identified DCAF16 as the responsible ligase for this activity. DCAF16 KO in Jurkat cells also abolished degradation and corroborated it as the responsible ligase. To assess if the activity is again based on monovalent gluing of BRD4 to DCAF16, the authors made RCS-03-017 (Figure 27C) with a minimal acrylamide moiety, and showed it retained similar degradation properties and maintained its dependency on DCAF16.

The complex structure with MMH2 (Figure 27D) indicated electrophiles off-of the JQ1 amide position might engage with Cys 173 or 178 in DCAF16 (although C173 is on a flexible loop and is not modeled in the structure). To identify the target cysteine the authors re-expressed various DCAF16 mutants in DCAF16 KO cells. WT and C58S restored degradation with STT-03–053–1 while C173S failed to restore it, suggesting that it may be the target covalent bond site.

The authors then tested a small SAR collection of electrophile positioning for HiBiT-BRD4 degradation at 5 h, a condition in which STT-03–053–1 is still active (DC_50_ = 2.6 μM; *D*_max_ = 30%) but RCS-03–017 is no longer active. Of the four electrophiles RCS-03–104–1–4 (Figure 27C), only the cyanoacrylamide showed degradation with DC_50_ = 80 nM and *D*_max_ = 70%. Varying the linker length between JQ1 and the cyanoacrylamide electrophile did not have large effects on degradation activity (DC_50_ ranged 53–95 nM and *D*_max_ 36–72%). Compounds with a rigid piperazine linker did not induce degradation suggesting that linker flexibility is required for BRD4 degradation by these compounds.

Proteomic experiments showed that BRD4 is still the main target for RCS-03–104–4. However, when tested in DCAF16 KO cells, it maintained its activity, suggesting it utilizes an alternative mechanism to mediate degradation. A CRISPR screen identified DCAF11 as the likely new ligase. Western blotting corroberated DCAF11 knockout prevented RCS-03–104–4-induced BRD4 degradation. The more sensitive HiBiT-BRD4 degradation assay showed that RCS-03–104–4 retained modest BRD4 degradation activity (*D*_max_ = 28%) likely through DCAF16. The authors used a BRET assay to show RCS-03–104–4 induces proximity between DCAF11 and BRD4. JQ1 was able to abolish this proximity, and none of the nondegrading RCS-03–104 series was able to show a similar proximity, further strengthening DCAF11 as the potential mediator of degradation.

Relatedly, Tin et al.^197^ synthesized a small collection of JQ1 linked to a cyanoacrylamide to explore BRD4 degradation. Compound **8b** (Figure 27C) showed robust degradation of BRD4 (DC_50_ = 305 nM; *D*_max_ ∼ 75%) and BRD3. Notably, analogues bearing the corresponding acrylamide or noncovalent propanamide were inactive.

CRISPR screening in a BRD4 reporter cell line identified among the top hits members of the CRL4-DCAF11 complex. DCAF11 KO in KBM7 cells rescued **8b** mediated BRD4 degradation. Reconstitution of WT DCAF11 could reinstate degradation, but not the triple mutant C443A/C460A/C485A^129,134^ suggesting **8b** binds similarly to previously reported DCAF11 PROTACs. The latter DCAF16/11 recruiters might be considered bifunctionals rather than glues, but presenting them here might put them in the broader context of DCAF16 mediated degradation of BRD4.

Lim et al.^198^ also tried to decorate JQ1 with various electrophiles. They synthesized 18 electrophilic JQ1 derivatives, and tested them for BRD4 degradation. Only one compound ML-1–50 (Figure 27C) showed degradation (DC_50_ ∼ 10 nM). The degradation was sensitive to proteasome and neddylation inhibition, and relatively selective as assessed by global proteomics.

To identify the ligase mediating this degradation, the authors synthesized a minimal alkyne derivative of the eletrophile, and performed a competitive pull-down proteomics experiment in which excess ML-1–50 (200 μM) competed over a 100 of the alkyne’s proteomic targets, only one of which was an E3 ligase, DCAF16.

An ABPP experiment, also identified C119 of DCAF16, but with a low competition ratio of 1.4, and numerous off-targets. LC/MS/MS of tryptic digested DCAF16 supported C119 as the site of modification.

BRD4 degradation by ML-1–50 was strongly reduced in DCAF16 KO cells. Re-expression of FLAG-DCAF16 WT restored the degradation, as did expression of DCAF16-C58S. However, re-expression of DCAF16-C119S did not restore degradation, further supporting yet another target cysteine site on DCAF16. The authors showed that their previously reported BRD4 degrader^199^ with a fumarate decoration (see section 3.4 below) is not mitigated by DCAF16 knockout, but is attenuated by RNF126 knockout, whereas ML-1–50 mediated BRD4 degradation is not affected by RNF126 KO.

To assess the scope of the vinyl sulfonamide piperazine ability to recruit DCAF16, the authors transplanted it onto various ligands. Installing it on ribociclib^200^ resulted in CDK4 degradation in the 5–10 μM range, which was mitigated in DCAF16 KO cells. The degradation was relatively selective with a handful of off-targets. Installing the vinyl sulfonamide on a SMARCA2/4 binder resulted in strong degradation in MV-4–11 cells, and weaker in HEK293, the latter was abolished on DCAF16 KO. Similar results were obtained with binders of AR, BCR-ABL and BTK, for the latter two degradation was demonstrated to diminish on DCAF16 KO of KD.

### 3.4 RNF126

**Figure 28:**
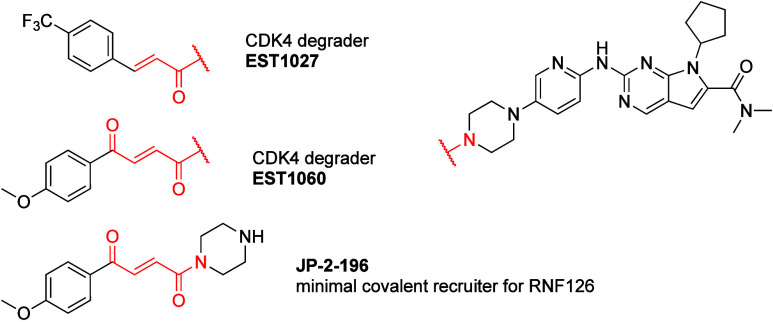
RNF126 covalent recruitment handles. Chemical structures of covalent handles that can be installed on ligands to turn them into recruiters of RNF126 E3 ligase. In this example, installation on ribociclib turned it into a CDK4 degrader.

Toriki et al.^199^ were looking for small chemical modifications that might convert a small molecule binder into a degrader. As a model system they used the CDK4/6 inhibitor ribociclib. They synthesized nine analogues of ribociclib, one of which, EST1027 (Figure 28), showed >50% degradation of CDK4 but not CDK6. Proteomics identified 100 additional targets down regulated along with CDK4 (but these may be downstream effects). The reduced version of EST1027 could not degrade CDK4, suggesting a requirement for covalent binding.

Transferring the cinnamamide moiety to another CDK4/6 inhibitor, palbociclib, did not result in degradation. The authors turned to optimize the degradation handle and made seven additional electrophilic analogues. One analogue EST1060 (Figure 28), was able to degrade CDK4 based on palbociclib as well (although still no CDK6 degradation).

isoDTB-ABPP experiments with EST1027 identified ∼50 proteins with competition ratios >2. Of these, the authors focused on C32 of RNF126 (the only UPS related target). This cysteine is one of four cysteines in a zinc coordinating site.

RNF126 knockdown, abrogated EST1027-mediated CDK4 degradation further suggesting this is the ligase recruited for degradation. EST1027 treatment also resulted in some degradation of RNF126 itself. LC/MS/MS analysis of tryptic digests following treatment with EST1027 or EST1060 also pointed to C32 as the site of modification.

To find the minimal pharmacophore required to covalently interact with RNF126 the authors assessed covalent binding (by in-gel ABPP competition) of various truncations of EST1027 and EST1060 that all contain the electrophile. This revealed the piperazine derivative JP-2–196 (Figure 28), drives most of the binding. A pull-down proteomics experiment with an alkyne derivative of this minimal recruiter identified RNF126 among 110 other targets including five other E3 RING ligases.

Transplanting the p-Methoxy-fumarate motif onto ligands that already contain piperazines or morpholines showed the resulting covalent derivatives are able to degrade the targets: BCR-ABL, PDE5, SMARCA2/4 and LRRK2 in the 1–10 μM range. They were also able to install this moiety on ligands not originally containing a piperazine such as ligands for BRD4, BTK, HDAC1/3, and an AR-V7 ligand. These led to μM potency degraders for all (except for BRD4 that showed nM potency).

### 3.5 UBE2D-NFκB

King et al.^201^ set out to discover covalent molecular glues. They screened a library of 750 cysteine-reactive covalent ligands for antiproliferative effects in HAP1 cells. This resulted in three reproducible hits reducing proliferation by >90%. To assess if these mediate their effect through a Cullin E3 ubiquitin ligase they tested the hits, as well as positive control dCeMM1^202^ in a UBE2M KD cell line (lacking neddylation). This knockdown, as well as neddylation and proteasome inhibitors showed a mild rescue of the effects by EN450 (Figure 16A) and dCeMM1.

An isoTOP-ABPP experiment with EN450 found 81 targets with a competition ratio of >1.3. This is a very low threshold corresponding to low occupancy, but the authors reasoned that if the binding is to a component of the UPS, partial occupancy should suffice to mediate function. The only UPS component identified was C111 of the ubiquitin-conjugating enzyme E2D (UBE2D). This is not the catalytic cysteine of this enzyme (which is C85). An alkyne derivative of EN450, still had antiproliferative effects that were sensitive to neddylation inhibition. The alkyne was able to label UBE2D C85A, and to a lesser extent C111A, suggesting at least some of its labeling was mediated by C111.

There are four isoforms of UBE2D (1–4) that could not be distinguished by proteomics. KD of UBE2D1, UBE2D4, or all four isoforms diminished a little the antiproliferative effects, while KD of UBE2D2 or UBE2D3 sensitized the cells even more to EN450.

To identify the potential degradation target, the authors used global proteomics comparing treatment with EN450 (50 μM; 24 h) with DMSO and found a single target that was downregulated by more than 4-fold, the p105 subunit of NFκB1. This was confirmed by WB. The reduced (noncovalent) version of EN450 did not affect NFκB1 levels.

*In vitro* experiments with purified UBE2D1 and GST-NFκB1 showed that EN450 induces a stronger interaction between the two proteins in a pull-down experiment and induces more ubiquitination of NFκB1 in a ubiquitination assay. To try and link NFκB to the antiproliferation phenotype, the authors overexpressed it and showed it slightly defends against the compound in two cell lines.

This study has clear limitations in terms of the potency and selectivity of EN450, as well as the magnitude of the phenotypes (as acknowledged by the authors) and follow up optimization should clarify the mechanism and its contribution to the antiproliferative effects.

### 3.6 UBR7-p53

**Figure 29:**
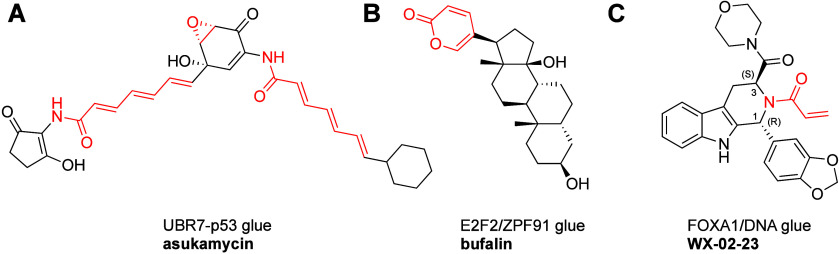
Various examples of covalent molecular glues. (A) A natural product covalent glue of UBR7 and p53. (B) A natural product molecular glue degrader of E2F2 recruiting ZPF91. (C) A covalent glue that stabilizes DNA interaction with the transcription factor FOXA1.

Isobe et al.^203^ investigated the poly electrophilic natural product asukamycin (Figure 29A) in the context of triple negative breast cancer. They showed it has low-μM antiproliferative activity in 231MFP cells. Using isoTOP-ABPP they identified C374 of the E3 ligase UBR7 as the top target for this compound. Knockdown of UBR7 in these cells did not affect proliferation, but conferred resistance to asukamycin, suggesting asukamycin does not inhibit the protein’s function. Re-expression of WT UBR7 in KD cells resensitized them to asukamycin, but not re-expression of a C374A mutant.

To identify potential new interaction partners mediated by asukamycin binding, the authors performed FLAG pulldowns on FLAG–UBR7-expressing cells. This resulted in eight UBR7 and compound specific enriched proteins. The authors focused on two targets: p53 and PRKDC (DNA protein kinase). These targets were validated by pulldown-WB experiments, which also identified a higher molecular mass band that may correspond to the tranary UBR7-asukamycin-p53 complex. The band was treatment specific and was attenuated on UBR7 knockdown.

Knockdown of p53, (but not of PRKDC) rescued the cells from asukamycin antiproliferative effects. To try and further understand the mechanism, the authors show that asukamycin leads to thermal stabilization of p53 in cell lysates, and increased p53 binding to its consensus DNA when p53 was added to lysates. Consistent with these observations asukamycin increased the expression of several p53 target genes and a p53 reporter, which were all reduced following UBR7 knockdown. All of these, suggesting that the ternary complex activates p53 to lead to the antiproliferative effects.

A close analogue of asukamycin, manumycin A, showed similar effects (formation of high-molecular weight band, increasing p53 activity, pull-down of p53 by FLAG–UBR7), but upon reducing its epoxide moiety, it lost all of these activities.

While several questions remain open following this study (such as where does UBR7 bind on p53 and how does that stabilize/activate it) it does demonstrate the gluing capacity of poly electrophilc natural products, and suggests that in the future we might be able to design synthetic bielectrophilic covalent glues.

In this regard it is worth mentioning a recent study from the Gray group^204^ where they installed two electrophiles on kinase inhibitors, as a strategy to avoid sensitivity to resistance mutations in the nucleophilic residue (often witnessed in oncology for targeted covalent inhibitors). While not a molecular glue, it shows synthetic bidentate electrophiles can be designed to target pairs of nucleophiles and in the future this might be applied to protein–protein interfaces.

### 3.7 ZPF91-E2F2

Liu et al.^205^ characterized the natural product bufalin (Figure 29B). After demonstrating its viability effect in HepG2 cells, they synthesized a biotinylated derivative to facilitate its target discovery. With this probe they screened a human proteome microarray, to find the transcription factor E2F2 as the top hit. The binding was validated by a pull-down assay and microscale thermophoresis (MST; *K*_D_ = 3.15 μM). Truncation studies mapped the interaction to the DNA binding and dimerization domains of E2F2. WB analysis showed that E2F2 levels are depleted following treatment with bufalin (DC_50_ < 400 nM). The degradation was proteasome dependent and bufalin induced ubiquitination of E2F2. To identify the potential effector, the authors used Co-IP/MS. Out of hundreds of enriched partners, only two were E3 ligases, of which ZPF91 was validated by WB. KD of ZPF91 reduced bufalin mediated degradation and ubiquitination. MST showed bufalin bound ZPF91 (*K*_D_ = 462 nM) and biotin-bufalin could pull it down from cells. LC/MS/MS analysis suggested C349 as a possible modification target on ZPF91, and a C349A mutant showed much weaker pull-down than WT. The α-pyrone group alone was able to bind ZPF91 by MST. Pull-downs with biotin–bufalin showed the α-pyrone blocked pull-down of ZPF91 and androsterone blocked pull-down of E2F2. Both substructures suppressed E2F2 degradation. Supporting this ability to bind both partners separately, E2F2 degradation with bufalin showed a hook effect at higher concentrations. In this regard bufalin acts like a bifunctional degrader (with a minimal/no linker).

### 3.8 FOX1A-DNA

In a recent preprint^206^ the Cravatt group reports a new type of covalent glue that mediates protein–DNA interaction. Using isoDTB-ABPP in 22Rv1 cells they profiled four tryptoline acrylamide stereoprobes and identified stereoselective and proteomically selective binding of WX-02–23 (Figure 29C) to C258 of the transcription factor FOXA1. A stereopair of alkyne analogues validated this result in complementary competitive pull-down experiments. Both the stereoselectivity, as well as SAR around a couple of close analogues suggested binding is driven by specific molecular recognition. Homology modeling suggested that C258 is located on a dynamic loop located near the protein–DNA interface.

The authors expressed an MBP-FOXA1 fusion that maintained stereoselective binding to their alkyne entantiopair, in a C258 selective manner in HEK293 cells. The purified construct, however, lost the stereoselective binding. Adding the purified protein to nuclear lysate restored its stereoselective binding, while treatment with the nuclease Benzonase abolished it, altogether suggesting the stereoselective binding might be DNA dependent. Indeed, addition of a 31 base pair DNA duplex with FOXA1 canonical binding sequence to the purified protein restored stereoselective and site-specific binding of the alkyne probe. Pretreatment with WX-02–23 stereoselectively blocked alkyne binding (in the presence of DNA) with IC_50_ = 8 μM.

The authors used two approaches to show the compound induces FOXA1 DNA binding in lysates. One based on luciferase-tagged FOXA1 binding to DNA fixed onto streptavidin coated plates, and another using NanoBRET between NanoLuc tagged FOXA1 and a TAMRA-tagged DNA. Both methods significantly showed about a 2-fold increase of DNA binding, in a stereoselective manner that is dependent on C258. The EC_50_ for increasing DNA binding for FOXA1 was 2 μM. In further DNA binding studies, the authors show that compound binding increased affinity for noncanonical sequences in a specific 3-base-pair region outside the canonical binding motif, therefore relaxing the overall binding preferences of FOXA1.

ChIP-Seq experiments following treatment with either of the stereoprobes did not show a change in the overall magnitude of FOXA1 binding to the genome, but rather, the active probe caused a redistribution of FOXA1 binding sites across the chromatin, both losing binding sites that were detected in DMSO treatment as well as gaining new binding sites. Further experiments showed that this redistribution of binding sites led to changes in chromatin accessibility.

To summarize this section, there are now examples for covalent molecular glues that bind one interaction partner covalently, create a new interface and stabilize the binding of the second interaction partner (14–3–3, NFκB). In other cases, the noncovalent binding is to one interaction partner, followed by covalent bond formation with the second protein after the noncovalent ternary complex is formed (K-Ras^G12C^, DCAF16) and yet in a third class the interaction of the glue with both partners is covalent (p53-UBR7).

Covalent glues can endow increased potency. For example, covalent 14–3–3 glues are superior compared to noncovalent, RMC-6291 is more potent than approved irreversible K-Ras^G12C^ drugs and MMH1/2 are very potent as monovalent BRD4 degraders. Covalent glues can also (like noncovalent glues) unlock challenging targets such as p53.

A recurring motif for the discovery of covalent glues is the installation of electrophilic moieties on known protein binders (Table 4) as we see for DCAF16, in more than one case, DCAF11, RNF126 and even in the case of the CypA/KRas^G12C^ interaction. This trend will likely increase in the near future as it has been very productive so far.

**Table 4:** Covalent Molecular Glues

effector	target	effect	discovery
14–3–3σ	various	target sequestration	direct electrophile screens
CypA	K-Ras^G12C^	inhibition	electrophilic decoration of ligand
DCAF16	BRD4	degradation	electrophilic decoration of ligand
RNF126	various	degradation	electrophilic decoration of ligand
UBE2D	NFκB	degradation	phenotypic screen
UBR7	p53	stabilization	natural product
ZPF91	E2F2	degradation	natural product
FOXA1	DNA	binding specificity modification	chemoproteomic screen

## 4 Summary and Outlook

**Figure 30:**
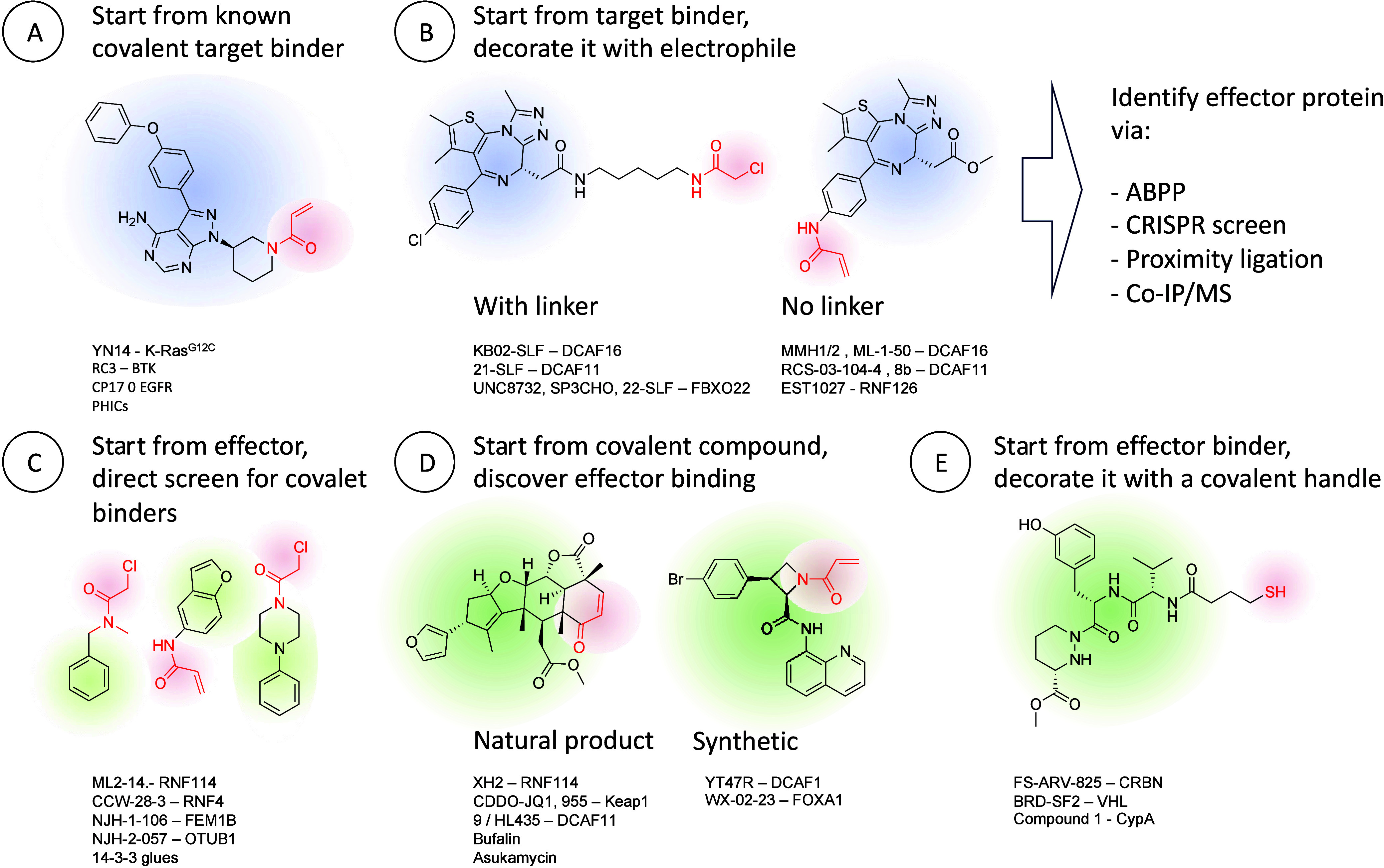
Strategies for the discovery of covalent proximity inducers.

The literature on covalent proximity inducers is prolific (I apologize for any missing report), and while reviewing it, several themes emerge repeatedly. First, are the routes of discovery of new covalent proximity inducers (Figure 30; Tables 2,4). The most straightforward strategy is to start with a known covalent binder for the target (section 2.1.1; Table 1; Figure 30A) which proved a productive strategy even in the absence of catalytic turnover. Next, decorating a known target binder, or effector binder with a covalent handle, with or without a linker, led to the discovery of several effectors through both bivalent and monovalent compounds (Figure 30B,E). This strategy typically necessitates identification of the covalent protein target through various methods. Direct screening of covalent binders against recombinant proteins (Figure 30C) was very productive, but typically requires extensive medicinal chemistry optimization of the discovered binders, otherwise the recruiters might be of lower quality (see below). Finally, chemoproteomic characterization of electrophilic natural products, or synthetic compounds that demonstrated direct cellular engagement with effector proteins unlocked several new effectors that could be recruited (Figure 30D). The two latter approaches also require optimization of the exit vector and linker length to the target ligand, but in the reviewed work typically less than five linkers were evaluated.

Another theme that repeats often is evidence of activity despite partial, and at times very low, occupancy of the effector protein in cells. Examples include 10% and 40% engagement with SLF and JQ1 DCAF16 PROTACs,^106^ 30% engagement of RNF4 with CCW-28–3,^119^ limited engagement of 21-SLF with DCAF11,^129^ 20% engagement of YT47R with DCAF1,^117^ 25% occupancy of EN67 with UBE2D,^144^ 7% occupancy of SKP1 for EN884,^147^ 30% and 20% modification of FBXO22 by SP3CHO^159^ and 22-SLF,^160^ respectively, and very low ABPP competition ratios for DUBTACs against OTUB1^162^ and ML-1–50 against DCAF16.^198^

While reports are accumulating on new E3-ligases being successfully recruited covalently for PROTACs, it is sobering to see there are now more effectors that were covalently recruited for degradation (13) than targets demonstrated to be degraded (**11**; Table 2) with BRD4 and FKBP12 dominating as model systems. More effort should be invested in demonstrating the generality of such recruiters, and therefore their utility. The reported covalent recruiters themselves are often promiscuous binders (sometimes by design) and require additional optimization that is rarely reported.

In this context of targeted degradation, the E3 ligases DCAF16, DCAF11, and recently FBXO22 were repeatedly discovered as mediators of degradation by electrophilic compounds in unbiased campaigns. Leading authors to hypothesize electrophilic quality control might be their endogenous role. In particular, the recent discovery of the intrinsic affinity of BRD4 to DCAF16^110,194^ makes it an immediate suspect for any JQ1-based electrophilic PROTACs. Counter-screening in DCAF16 and DCAF11 (and perhaps FBXO22) knockout cells should now become a standard control in covalent degrader reports. This should add to additional best practices for the development and characterization of covalent probes and PROTACs.^207,208^ The sensitivity to electrophiles of DCAF16/11 also suggests a lower reactivity, selective covalent recruiter might be developed to harness them for targeted degradation.

Another point worth considering in this regard is how subtle SAR around the electrophile, or in the placement of the electrophile can lead to completely different target engagement. For example compare RCS-03–017 targeting DCAF16 with RCS-03–104–4 and 8b targeting DCAF11, or compare SP3CHO with 22-SLF, both targeting FBXO22, but through different cysteine residues, or ML-1–50 and MMH2 both targeting DCAF16, but again through different cysteine residues. This begs extra scrutiny in the characterization of new covalent degraders.

From a chemical perspective, despite several recent attempts to target additional nucleophilic amino acids (lysine in 14–3–3, histidine and serine in CRBN and VHL, respectively) the overwhelming majority of covalent proximity inducers still target cysteine residues (Tables 2,4). It would seem this is an untapped opportunity for future proximity inducers discovery. Incorporating electrophiles against a wider scope of residues may expand the scope of effectors that can be recruited.

Several related topics were left out of the scope of this review, including peptide/protein based proximity inducers^209^ and covalent peptide/protein reagents as a whole,^210−214^ that may serve as a future avenue for innovation in the field.

Compared to the explosion in (noncovalent) PROTACs reported in the medicinal chemistry and chemical biology field, most of the covalent proximity inducers were discovered by a small number of groups, representing an opportunity for more research. One of the earliest papers reporting covalent PROTACs^106^ foreshadowed the future, noting that more than 562 cysteines on 211 E3 ligases have been detected in various ABPP experiments, and predicted: “The extent to which E3 ligase cysteines can be engaged by heterobifunctional, or even molecular glue, compounds to promote ligand-induced protein degradation should represent a fertile area of future investigation”. This prediction has certainly held true as evident by this review, and could now be extended beyond degradation to additional classes of covalent proximity inducers, a very fertile ground for future investigation.
